# Effectiveness of a Comprehensive Program Including a Novel Concentrated High-Protein, High-Calorie Oral Nutritional Supplement to Enhance Nutritional and Morphofunctional Recovery in Malnourished Patients with Cancer: The ONAVIDA Study

**DOI:** 10.3390/nu18091398

**Published:** 2026-04-29

**Authors:** José Manuel García-Almeida, Rocío Fernández-Jiménez, Ana Hernández-Moreno, Gabriel Olveira, Mercedes Vázquez-Gutiérrez, Carolina Dassen, Pedro Pablo García-Luna, Amalia González-Jiménez, Josefina Olivares, María García-Duque, Mª José Martínez-Ramírez, Juan Manuel Guardia-Baena, María I. Rebollo-Pérez, Miguel Civera, Visitación Álvarez-de Frutos, Vicente Faus, Lucía Díaz-Naya, José Joaquín Alfaro-Martínez, Alejandro Sanz-París

**Affiliations:** 1 Department of Endocrinology and Nutrition, Hospital Clínico Universitario Virgen de la Victoria, 29010 Málaga, Spain; jgarciaalmeida@gmail.com (J.M.G.-A.); rociofernandeznutricion@gmail.com (R.F.-J.); 2 IBIMA BIONAND (Instituto de Investigación Biomédica de Málaga), 29590 Málaga, Spain; gabrielm.olveira.sspa@juntadeandalucia.es; 3 Department of Medicine and Dermatology, Málaga University, 29016 Malaga, Spain; 4 Department of Endocrinology and Nutrition, Quironsalud Málaga Hospital, Av. Imperio Argentina, 29004 Malaga, Spain; 5 Centro de Investigación Biomédica en Red de Fisiopatología de la Obesidad y Nutrición (CIBEROBN), Instituto de Salud Carlos III, 28029 Madrid, Spain; 6 Department of Endocrinology and Nutrition, Hospital Universitario de Navarra, C. de Irunlarrea, 3, 31008 Pamplona, Spain; ahmoreno@alumni.unav.es; 7 Department of Endocrinology and Nutrition, Hospital Regional Universitario de Málaga, 29010 Málaga, Spain; 8 Centro de Investigación Biomédica en Red de Diabetes y Enfermedades Metabólicas Asociadas (CIBERDEM), Instituto de Salud Carlos III, 29010 Málaga, Spain; 9 UGC de Endocrinología, Nutrición y Riesgo Vascular, Hospital Torrecárdenas, C. Hermandad de Donantes de Sangre, 04009 Almería, Spain; mmervg@gmail.com; 10 Department of Endocrinology and Nutrition, Health Research Institute Fundación Jiménez Díaz, Av. de los Reyes Católicos, 2, 28040 Madrid, Spain; carodassen@gmail.com; 11 Unidad de Gestión Clínica de Endocrinología y Nutrición, Instituto de Biomedicina de Sevilla (IBiS), Hospital Universitario Virgen del Rocío, Consejo Superior de Investigaciones Científicas (CSIC), Universidad de Sevilla, 41013 Seville, Spain; garcialunapp@yahoo.es; 12 Department of Endocrinology and Nutrition, Unidad de Nutrición Clínica y Dietética, Hospital Universitario San Cecilio, Av. del Conocimiento, 18007 Granada, Spain; amalia@ugr.es; 13 Endocrinology and Nutrition Department, Hospital Universitario Son Llàtzer, Health Research Institute of the Balearic Islands (IdISBa), Ctra Manacor km 4, 07198 Palma de Mallorca, Spain; jolivares@ssib.es; 14 Servicio de Endocrinología y Nutrición, Complejo Asistencial Universitario de León, Gerencia de Salud de Castilla y León (SACYL), Calle Altos de Nava, 24008 León, Spain; mariagduque@saludcastillayleon.es; 15 Endocrinology and Nutrition Service, Jaen University Hospital, Avenida del Ejército Español, 10, 23007 Jaén, Spain; mjmramirez56@gmail.com; 16 Department of Endocrinology and Nutrition, Virgen de las Nieves University Hospital, Av. de las Fuerzas Armadas, 2, Beiro, 18014 Granada, Spain; guardiabaena@gmail.com; 17 Endocrinology and Nutrition Service, Hospital Juan Ramón Jiménez, Ronda Norte, 21005 Huelva, Spain; misabel.rebollo.sspa@juntadeandalucia.es; 18 Service of Endocrinology and Nutrition, Hospital Clinico Universitario of Valencia, Av. de Blasco Ibáñez, 17, El Pla del Real, 46010 Valencia, Spain; mi.civeraa@comv.es; 19 Department of Medicine, University of Valencia, Av. de Blasco Ibáñez, 17, El Pla del Real, 46010 Valencia, Spain; 20 Service of Endocrinology and Nutrition, Hospital Universitario de Guadalajara, C. Donante de Sangre, 19002 Guadalajara, Spain; vvad03@gmail.com; 21 Department of Pharmacy, Hospital Costa del Sol, A-7, Km 187, 29603 Marbella, Spain; vicente.faus.sspa@juntadeandalucia.es; 22 Department of Endocrinology and Nutrition, Hospital Universitario de Cabueñes, Los Prados, 395, 33394 Gijón, Spain; ldnaya@live.com; 23 Complejo Hospitalario Universitario de Albacete, c/Hermanos Falcó, 37, 02008 Albacete, Spain; jalfaro@sescam.jccm.es; 24 Endocrinology Department, University Hospital Miguel Servet, Paseo de Isabel la Católica, 1-3, 50009 Zaragoza, Spain

**Keywords:** malnutrition, cancer, novel concentrated oral nutritional supplement, morphofunctional assessment, nutritional status, nutritional ultrasound, bioelectrical impedance

## Abstract

Background/Objectives: Malnutrition in cancer adversely affects treatment outcomes and survival. Early intervention through oral nutritional supplements (ONSs) and dietary counseling can improve outcomes. This study evaluated the evolution of nutritional and morphofunctional parameters over three months in malnourished patients with cancer undergoing a comprehensive nutritional support program comprising dietary counseling, physical activity, and a novel concentrated high-protein, high-calorie ONS (cHPHC-ONS) with a high intrinsic leucine content. Methods: A prospective, observational, multicenter cohort study was conducted across 18 public hospitals in Spain. Two hundred thirty malnourished patients with cancer were enrolled: 147 naïve (no ONS treatment in the last three months) and 83 non-naïve (who transitioned to cHPHC-ONS after inadequate response to initial ONSs). Nutritional status was assessed using Global Leadership Initiative on Malnutrition (GLIM) criteria and morphofunctional parameters via bioelectrical impedance analysis, nutritional ultrasound, handgrip strength, the Timed Up and Go (TUG) test, and analysis of biochemical parameters. Results: After three months, 23.8% achieved normal GLIM nutritional status (*p* < 0.0001), with a greater improvement seen in non-naïve patients (28.4%, *p* < 0.0001). Weight loss ceased in 42.6% (*p* < 0.0001). and inflammation resolved for 10.3% (*p* = 0.0015). Non-naïve patients experienced a significant increase in fat-free mass index (*p* = 0.0159), appendicular skeletal muscle index (*p* = 0.0248), and rectus femoris cross-sectional area (*p* = 0.0016). Muscle strength increased significantly by +1.7 kg (*p* = 0.0025), and TUG test time decreased by 1.13 s (*p* = 0.0003) overall. Conclusions: The comprehensive nutritional support program—including a novel cHPHC-ONS, along with dietary and physical activity guidance—significantly improved the nutritional and morphofunctional status of malnourished patients with cancer, with benefits particularly evident in non-naïve individuals. Limitations: Observational design, no control group, short follow-up, and unadjusted non-multivariable comparisons, limiting causal inference.

## 1. Introduction

Cancer remains a major global health challenge, with incidence and mortality rates expected to rise significantly [1]. In 2022, GLOBOCAN reported around 20 million new cancer cases and over 9.7 million deaths worldwide [2]. In Spain, the burden is substantial, with 290,371 new cancer cases and 113,000 cancer-related deaths reported the same year [3]. As cancer prevalence grows, nutrition-related complications have become a critical concern, as both the disease and its treatments often lead to a decline in nutritional status [4].

Malnutrition is a frequent and clinically impactful complication in cancer, affecting 16.0–71.6% of patients [5]. It results from tumor-related metabolic changes, treatment side effects, and psychological distress, leading to reduced intake, increased energy demands, and progressive nutritional decline [4,6,7,8]. Associated outcomes include weight and muscle loss, cachexia, poorer treatment tolerance, and reduced survival [4,9,10,11].

The Global Leadership Initiative on Malnutrition (GLIM) has established internationally recognized consensus-based criteria for diagnosing malnutrition in adults across diverse medical conditions [12,13,14]. Since its introduction in 2019, GLIM has reshaped the diagnostic paradigm by promoting a standardized, clinically relevant approach that balances diagnostic accuracy with practical applicability [12,13,14]. The framework requires the presence of at least one phenotypic criterion—such as unintentional weight loss, low body mass index (BMI), or reduced muscle mass—and one etiological criterion, including reduced dietary intake or evidence of inflammation (e.g., elevated C-reactive protein) [12,13,14]. Notably, the inclusion of inflammation as an etiological criterion and muscle mass as a phenotypic criterion highlights the necessity of evaluating muscle mass and the importance of morphofunctional assessment in clinical practice. In this context, techniques such as bioelectrical impedance analysis (BIA), nutritional ultrasound (both for muscle quantity and quality assessments), dynamometry (for muscle strength assessments), and functional tests have become essential for a comprehensive evaluation of body composition and functional status in malnourished individuals [15]. These techniques allow early detection of malnutrition and enable the initiation of timely strategic interventions that can significantly improve patient outcomes [16].

Global clinical guidelines, including European Society for Clinical Nutrition and Metabolism (ESPEN) guidelines and expert consensus, emphasize the importance of routine nutritional screening and early intervention, recommending dietary counseling for all patients with cancer regardless of their baseline nutritional status or cancer stage [1,17,18]. ESPEN guidelines advise that patients affected by cancer should receive 25–30 kcal/kg/day and up to 1.5 g/kg/day of protein to support optimal nutritional status [1,18]. Oral nutritional supplements (ONSs) are advised as an adjunct to dietary counseling to help achieve nutritional goals and support treatment, particularly during chemotherapy, where weight stabilization is associated with better survival outcomes [1,19,20,21,22]. ONSs are typically rich in protein, energy, vitamins, and minerals, and they are useful to compensate for deficiencies caused by disease and treatment [23,24,25,26]. Recent evidence consistently supports the role of ONSs in improving nutritional status, muscle mass, and functional outcomes in malnourished patients with cancer. Of note, ONSs not only mitigate treatment-related adverse effects but also potentially enhance therapy efficacy [27,28,29]. Studies have shown that ONSs can preserve body composition, prevent fat accumulation, improve muscle mass, and maintain biochemical stability during chemotherapy [30,31,32].

Compliance with ONSs remains a major challenge in patients with disease-related malnutrition (DRM) and cancer, often due to poor appetite, large required volumes, gastrointestinal intolerance, and unappealing taste or texture [33]. Energy-dense formulations (>2.0 kcal/mL) with varied flavors can enhance adherence by reducing the volume needed and accommodating individual preferences [34]. A recent real-world study introduced a novel concentrated high-protein, high-calorie ONS (cHPHC-ONS; ≥2.1 kcal/mL, 32 g protein/200 mL, ~3.6 g intrinsic leucine) combining fast-absorbing whey with slow-digesting casein proteins to address barriers to ONS compliance and enhance nutritional intake and clinical outcomes in patients with DRM [35]. The supplement was highly effective and well-tolerated, leading to better adherence and outcomes. After three months, patients had significantly less weight loss (from −6.75% to 0.5%, *p* < 0.01), reduced malnutrition rates (from 93.4% to 78.9%, *p* < 0.01), and enhanced muscle mass and function (increased appendicular skeletal muscle index and rectus femoris muscle area) [35]. Only 3.1% reported mild gastrointestinal symptoms, and compliance was high, with most patients following the regimen [35].

The present study aimed to assess the evolution in functional, nutritional, and body composition status in malnourished patients with cancer (stratified as naïve and non-naïve) undergoing a three-month comprehensive nutritional support program that included a novel cHPHC-ONS with a high intrinsic leucin content, dietary counseling, and physical exercise using morphofunctional and nutritional assessments.

## 2. Materials and Methods

### 2.1. Study Design and Eligibility Criteria

This observational, prospective, multicenter cohort study was conducted between 19 September 2023 and 29 October 2024 across 18 public hospitals in Spain.

The study included adult (≥18 years) non-hospitalized patients with cancer diagnosed with malnutrition according to the GLIM criteria [14] who were deemed by their treating clinician to have elevated energy and protein requirements and who provided written informed consent. The enrolled patients were divided into two cohorts. Cohort 1 comprised naïve patients (those who had either never received ONSs or had not received them within the previous three months) who required a cHPHC ONS due to elevated nutritional needs. Cohort 2 comprised non-naïve patients who had previously received ONSs but were unable to meet their nutritional objectives, necessitating their replacement with a cHPHC ONS.

Patients were excluded if they had an acute medical condition at enrollment (excluding minor surgical procedures), a diagnosis of pancreatic cancer, were receiving palliative care and/or had an Eastern Cooperative Oncology Group (ECOG) performance status greater than 3, were undergoing chronic corticosteroid therapy, had poorly controlled type 1 or type 2 diabetes mellitus, acute or severe chronic kidney disease, severe hepatic impairment, advanced organ failure, or malabsorption syndromes associated with impaired metabolism. Additional exclusion criteria included pregnancy, inability to complete the study questionnaires, or participation in another nutrition-related clinical study during the observational period.

### 2.2. Nutritional Intervention

The enrolled patients participated in a comprehensive nutritional support program consisting of three key components: (1) supplementation with a novel cHPHC-ONS providing ≥2.1 kcal/mL, 32 g of protein per 200 mL, with a combination of high-quality whey protein and casein, and around 3.6 g of intrinsic leucine per bottle (Nestlé Health Science, Vevey, Switzerland) (Supplementary Table S1). For the present study, most patients received 2 bottles per day (77.3%), followed by 1 bottle per day (17.0%). A smaller proportion received 3 bottles per day (5.24%), while only one patient (0.4%) received 4 bottles per day; (2) individualized dietary recommendations, carefully tailored to each patient’s unique nutritional requirements, focusing on diets enriched with protein and calories from high-value nutrients to enhance tolerance and absorption; and (3) a structured, multicomponent exercise program incorporating warm-up exercises, progressive muscle-strengthening trainings for major arm and leg muscles (adjusted weekly based on strength and performance evaluations), and individualized cardiovascular exercises to maximize muscle mass and functional capacity. The nutritional support program has been developed and validated based on a previous study by García Almeida et al. [36] (Supplementary Material File S3).

The decision to prescribe cHPHC-ONS was made by the participating physicians, based solely on their clinical judgment and within the framework of routine clinical practice. In this study, and consistent with standard practice, the follow-up period lasted three months.

### 2.3. Data Collection

**Figure 1:**
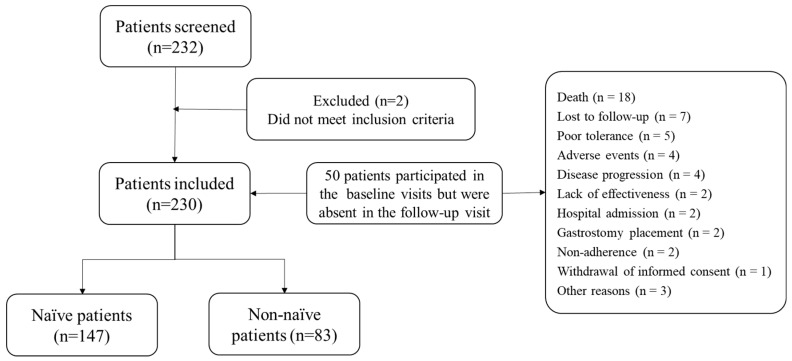
Patient screening, inclusion, and follow-up flow diagram.

Primary and secondary data were collected in accordance with standard clinical protocols, sourced from patients’ medical records and patient-reported outcome (PRO) questionnaires. All data were systematically recorded by the investigators in electronic case report forms (eCRFs). Prospective data collection started from the baseline visit (i.e., the date of patient enrollment, informed consent collection, and initiation of nutritional therapy) and continued through a three-month follow-up (±1 month) (Figure 1). Clinical and demographic data were recorded in the eCRF at both the baseline and final visits, along with PROs. Interim clinical data were extracted from routine follow-up documentation.

### 2.4. Objectives and Endpoints

This study aimed to assess the evolution in patients’ functional, nutritional, and body composition status through morphofunctional and nutritional assessments. All assessments were performed at baseline and at the three-month follow-up visit.

#### 2.4.1. Nutritional Assessment (GLIM Criteria)

Nutritional diagnosis was based on the GLIM criteria, which combine phenotypic and etiological factors to identify malnutrition. This comprehensive approach improved diagnostic accuracy and guided individualized interventions. Phenotypic criteria included weight loss (>5% in the last six months or >10% in the last six months), low BMI (<20 kg/m^2^ for <70 years or <22 kg/m^2^ for ≥70 years), and reduced muscle mass (e.g., fat-free mass index [FFMI] < 17 kg/m^2^ for men, <15 kg/m^2^ for women). Etiological criteria included reduced food intake and inflammation. Inflammatory status was assessed by measuring elevated levels of serum C-reactive protein (CRP), a well-established biomarker of systemic inflammation. As mentioned previously, muscle mass was assessed using BIA and other morphological techniques, such as nutritional ultrasound.

#### 2.4.2. Morphofunctional Assessment

##### Bioelectrical Impedance Analysis

BIA is a non-invasive technique used to assess body composition and cellular health by measuring the body’s resistance and reactance to electrical current [37,38]. It is particularly valuable in clinical nutrition for evaluating hydration status, muscle mass, and cell membrane integrity through the derived phase angle (PA), which serves as a prognostic marker in various disease states [37,38]. In this study, whole-body BIA was performed using a phase-sensitive single-frequency impedance analyzer (Whole Body Bioimpedance Vector Analyzer, Nutrilab, AKERN, Florence, Italy) operating at 50 kHz and 800 µA [39]. Patients were positioned supine with limbs slightly abducted. After skin cleansing with isopropyl alcohol, Ag/AgCl electrodes were placed on the right hand and foot. Measurements were taken after a five-minute rest. A comprehensive set of parameters was recorded, including resistance (Rz), reactance (Xc), and phase angle (PA, °) calculated as ([Xc/Rz] × (180 o/π), standardized phase angle (SPA, °), body cell mass (BCM, kg), fat mass index (FMI, kg/m^2^), FFMI (kg/m^2^), and appendicular skeletal muscle index (ASMI, kg).

##### Nutritional Ultrasound Measurement

**Figure 2:**
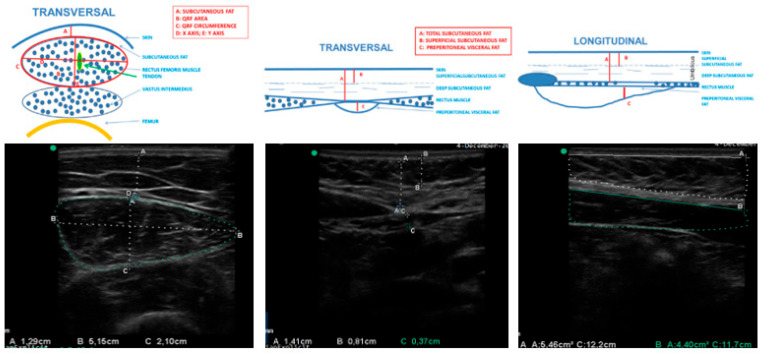
Ultrasound transverse and longitudinal views of the QRF muscle and abdominal regions. This figure illustrates the anatomical areas used for measurement, highlights the precise placement of the ultrasound transducer, and provides both an image and a schematic diagram of relevant anatomical structures. The upper panel shows anatomical landmarking, probe positioning, and representative transversal and longitudinal ultrasound images of the QRF, with measurements of subcutaneous fat and muscle dimensions. The lower panel shows standardized abdominal landmarking and ultrasound images illustrating measurements of subcutaneous fat layers, rectus abdominis muscle thickness, and preperitoneal (visceral) fat depth. Source: own elaboration.

Ultrasound imaging was used to assess muscle architecture, making it a practical tool for evaluating nutritional status and sarcopenia. It allowed for the measurement of muscle thickness, cross-sectional area, and muscle quality, which are critical indicators of functional capacity and prognosis [37,38]. In this study, a 10–12 MHz probe with a multifrequency linear array (Mindray Z60, Madrid, Spain) was used to assess the rectus femoris muscle of the quadriceps. The probe was placed perpendicular to the muscle at the lower third between the anterior superior iliac spine and the patella. Parameters measured included rectus femoris cross-sectional area (RF-CSA), quadriceps rectus femoris circumference (RF-CIR), rectus femoris horizontal axis (RF-X), rectus femoris vertical axis (RF-Y), and subcutaneous adipose tissue (L-SAT). All parameters were measured thrice, and the mean was recorded (Figure 2).

For the assessment of abdominal adipose tissue, ultrasound measurements were performed at the midpoint between the xiphoid process and the umbilicus. This method allowed for quantification of total subcutaneous adipose tissue (T-SAT) and preperitoneal adipose tissue (PAT) [40].

##### Functional Parameters

From a methodological perspective, dynamometry, particularly handgrip strength testing, is a widely accepted method for assessing muscle function and nutritional status [37,38]. In this study, handgrip strength was measured using a Jamar hydraulic hand dynamometer (Asimow Engineering Co., Los Angeles, CA, USA). Participants were seated with their elbows flexed at 90°, wrists in a neutral position, and feet flat on the floor. Three maximal isometric contractions were performed with the dominant hand, each lasting five seconds and separated by one-minute rest intervals. All parameters were measured thrice, and the mean was recorded in kilograms.

By comparison, the Timed Up and Go (TUG) test is a simple and reliable functional assessment used to evaluate mobility, balance, and fall risk, especially in patients with malnutrition. It reflects the patient’s ability to perform basic movements required for daily living [37,38]. In this study, participants were instructed to rise from a standard-height chair, walk three meters, turn around, return to the chair, and sit down. The total time taken to complete the task was recorded in seconds. The test was conducted under supervision to ensure safety and consistency.

##### Biochemical Parameters

Albumin, prealbumin, CRP, and the CRP/prealbumin ratio are recognized as clinically relevant biomarkers in patients with malnutrition [41], as they provide complementary information on nutritional status and systemic inflammatory response—two fundamental components of DRM. In the present study, these parameters were evaluated to characterize their association with patient outcomes and to elucidate the interaction between nutritional deficits and inflammation.

### 2.5. Statistical Methods

#### 2.5.1. Sample Size

A total of 230 patients were included in the study, comprising 147 individuals who had not previously received ONSs and 83 who had. This sample size was necessary to assess adherence to the nutritional program as a binary outcome. For dichotomic variables, such as adherence to the ONSs program, considering a 95% confidence interval and assuming a ratio of 0.5 (maximum indeterminacy), the resulting precision was ±0.081 for the cohort of ONSs naïve subjects, ±0.108 for the cohort of subjects who had previously received ONSs, and ±0.0647 for the total population.

#### 2.5.2. Statistical Analysis

All statistical analyses were performed using SAS^®^ version 9.2 (SAS Institute, Cary, NC, USA). Descriptive analyses were conducted to characterize both the qualitative and quantitative aspects of the collected data and to describe the study population. Continuous variables were summarized using means and standard deviations (SDs) or medians and ranges, as appropriate. Categorical variables were presented as frequencies and percentages, both for the total study population and relevant subgroups. For all variables, the number of observations (N) and the number of missing values (N missing) were reported.

The nutritional and morphofunctional status was reported for both naïve and non-naïve patients at the initial and final visits. Statistical significance (*p*-values) was assessed using the *t*-test or Wilcoxon signed-rank test for continuous variables, depending on their distribution, and the McNemar test for categorical variables.

## 3. Results

### 3.1. Patient Disposition

A total of 232 patients were screened for eligibility. Of these, two patients were excluded for not meeting the inclusion criteria, resulting in 230 patients (99.1%) who met all the inclusion criteria and were enrolled in the study. Among the enrolled participants, 147 (63.9%) were classified as naïve (Cohort 1) and 83 (36.1%) as non-naïve (Cohort 2) (Figure 1).

During the study period, 50 patients who completed the baseline visit did not attend the final follow-up visit. Reasons for discontinuation included the following: 18 died during the study, seven were lost to follow-up, four experienced adverse events, another four experienced disease progression, two experienced lack of effectiveness, two had hospital admissions, two following gastrostomy placement, two because of non-adherence, and one participant withdrew informed consent. Additionally, three patients were discontinued due to other unspecified reasons.

### 3.2. Baseline Demographic and Clinical Characteristics

The overall mean age (SD) of the study population was 64.38 (11.11) years. The mean age of naïve patients was 64.15 (11.36) years, whereas the mean age of non-naïve patients was 64.79 (10.71) years (*p* = 0.8087). In the total population, 40.2% were female, with a slightly higher proportion among the naïve group (44.0%) compared with the non-naïve (33.3%; *p* = 0.1491) group. BMI averaged 23.25 kg/m^2^ in the total population, with the naïve group having a mean BMI of 25.59 kg/m^2^ and the non-naïve group 22.66 kg/m^2^ (*p* = 0.1216) (Table 1).

The most prevalent cancer diagnosis among the study population was head and neck cancer, affecting 36.7% of patients, followed by gastric cancer (15.3%), colon and rectal cancer (14.0%), and lung cancer (13.1%). The other less frequent types of cancers included bladder, breast, endometrial/uterine, and ovarian cancers, as well as hematological malignancies such as lymphoma and leukemia. The distribution of cancer types was broadly similar between naïve and non-naïve patients (*p* > 0.05) (Table 1).

Regarding cancer staging, the majority of patients were diagnosed at advanced stages, with 37.1% at Stage IV and 31.2% at Stage III, while 22.6% were at Stage II and 9.1% at Stage I. The distribution was similar between the two cohorts, with a slightly higher proportion of Stage III cases in the non-naïve cohort (non-naïve: 34.6% vs. naïve: 29.3%) and Stage IV cases in the naïve cohort (naïve: 39.3% vs. non-naïve: 33.3%), although the differences were non-significant (*p* > 0.05). ECOG status indicated 30.9% were ECOG 0, 47.0% ECOG 1, and 22.2% ECOG ≥ 2. Notably, ECOG 2 was more frequent in naïve patients (23.8%) compared with non-naïve patients (12.1%), with statistical significance (*p* = 0.0308). Other ECOG categories did not differ significantly (*p* > 0.05). The most frequently reported comorbidities included chronic lung disease (8.3%), cerebrovascular disease (5.2%), congenital heart failure (4.8%), and uncomplicated diabetes mellitus (3.9%). The prevalence of comorbidities was similar between naïve and non-naïve patients (*p* > 0.05 for all parameters) (Table 1).

All patients (100%) met the GLIM diagnostic criteria for malnutrition. Weight loss was common, with 46.1% of patients reporting > 5% loss in the past six months and 46.5% reporting >10% loss during this same period. Low BMI was observed in 15.7% of patients (<20 kg/m^2^ if <70 years) and 9.6% (<22 kg/m^2^ if >70 years). Reduced muscle mass was highly prevalent (68.5%). Reduced food intake affected 83.9% of patients, and inflammation was present in 91.7%. No significant differences were found between naïve and non-naïve patients in GLIM nutritional criteria, corresponding to weight loss, low BMI, reduced muscle mass, reduced food intake, or inflammation (all parameters: *p* > 0.05) (Table 1).

The BIA revealed that the mean PA was 4.82° (SD 0.88) and SPA 0.37 (SD 1.23). The average BCM measured was 22.88 kg. Patients had a mean FMI of 5.69 kg/m^2^. The FFMI stood at 17.45 kg/m^2^, while the ASMI was recorded at 6.42 kg. These BIA parameters were also comparable between groups, with no statistically significant differences (all parameters: *p* > 0.05) (Table 1).

Muscle ultrasound showed an RF-CSA of 3.45 cm^2^ in the total population, with similar values in the naïve (3.53 cm^2^) and non-naïve (3.31 cm^2^) groups. The overall mean SAT was 0.87 cm, and it was significantly higher in naïve than in non-naïve (0.98 cm vs. 0.68 cm; *p* = 0.0109) patients. For RF-CIR, the total population averaged 8.89 cm, and it remained comparable between the naïve and non-naïve groups (8.97 cm vs. 8.76 cm, *p* = 0.852). The RF-X measurement followed a similar trend, with the total population averaging 4.29 cm and showing minimal difference between the naïve and non-naïve groups (4.41 cm vs. 4.11 cm, *p* = 0.4945). Regarding RF-Y measurements, on relaxation, it averaged 1.59 cm in the total population (naïve: 1.78 cm, non-naïve: 1.31 cm, *p* = 0.1127). On contraction, RF-Y values were slightly higher overall (1.84 cm). Naïve individuals demonstrated slightly higher RF-Y values compared with non-naïve individuals (2.01 cm vs. 1.60 cm, *p* = 0.4492) (Table 1).

Abdominal ultrasound in the total population showed mean T-SAT values of 1.67 cm, S-SAT of 0.81 cm, and PAT of 0.63 cm. T-SAT values slightly higher in naïve (1.71 cm) than in non-naïve (1.60 cm) patients. Similarly, S-SAT averaged 0.83 cm in naïve patients compared with 0.77 cm in non-naïve patients, while PAT was marginally lower in the naïve group, at 0.61 cm, compared with 0.67 cm in the non-naïve group (Table 1).

Functional assessments revealed an average handgrip strength of 27.42 kg and a mean TUG test duration of 10.32 s for the study population. When comparing the naïve and non-naïve groups, handgrip strength was comparable (*p* = 0.5287). However, non-naïve patients demonstrated significantly better functional mobility, as evidenced by a shorter TUG time versus naïve patients (9.41 s vs. 10.82 s; *p* = 0.0438) (Table 1).

Biochemical analysis in the total population revealed a mean albumin level of 3.92 g/dL, prealbumin 21.95 mg/dL, CRP 3.49 mg/L, and CRP/prealbumin ratio 0.20. Comparing the naïve and non-naïve groups, albumin was 3.95 g/dL versus 3.87 g/dL, prealbumin 21.46 mg/dL versus 22.78 mg/dL, CRP 3.32 mg/L versus 3.76 mg/L, and CRP/prealbumin ratio 0.18 versus 0.22, with no significant differences between the groups (all parameters: *p* > 0.05) (Table 1).

### 3.3. Evolution of Nutritional Status

**Figure 3:**
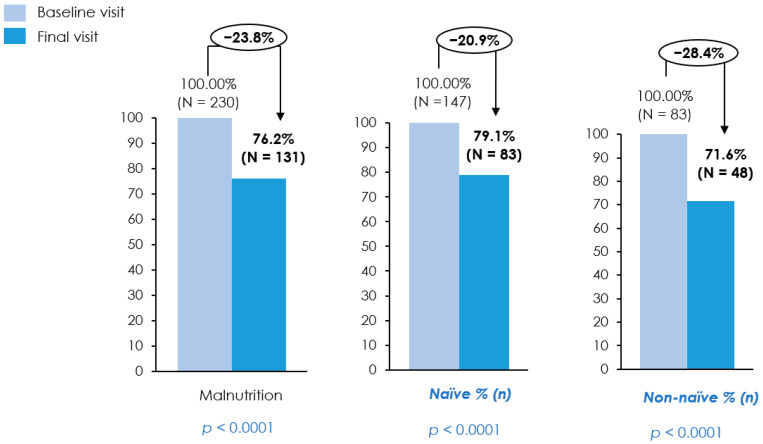
Prevalence of malnutrition according to the GLIM criteria at baseline and final visit. Bar charts present the proportion of patients classified as malnourished at baseline and at the final visit in the overall population, treatment-naïve patients, and non-naïve patients. Percentages and absolute numbers are shown above bars, with relative change indicated above each panel. Baseline and final visits were compared using a *t*-test or Wilcoxon signed rank test for continuous variables and the McNemar test for categorical variables; *p*-values are displayed. Additional Note: N refers to the number of malnourished patients. At baseline, the total number of patients was 230, while at the final visit, it was 182. Among naïve patients, 147 were evaluated at baseline and 111 at the final visit; among non-naïve patients, 83 were evaluated at baseline and 71 at the final visit. Percentages were therefore calculated based on the number of patients with available data at each specific time point.

At baseline, all patients met the GLIM criteria for malnutrition. By the final visit, 23.8% of patients achieved nutritional recovery based on the GLIM criteria, representing a clinically meaningful improvement in nutritional status (*p* < 0.0001) (Figure 3 and Table 2). This benefit was even greater among non-naïve patients, with 28.4% reaching nutritional recovery at follow-up, compared with 21% among naïve patients, underscoring the importance of nutritional interventions.

Significant improvements were observed in GLIM weight loss criteria across the overall population (*p* < 0.0001), as well as in the naïve and non-naïve cohorts (*p* < 0.0001). The proportion of patients with >5% weight loss in the last six months dropped from 46.1% to 27.5%, and those with >10% weight loss in the last six months decreased from 46.5% to 22.5% (Figure S1, Supplementary Material). The proportion of patients with low BMI remained relatively stable across time points in both age categories (<70 and ≥70 years), with no significant differences observed (*p* = 0.9109) (Table 2).

**Figure 4:**
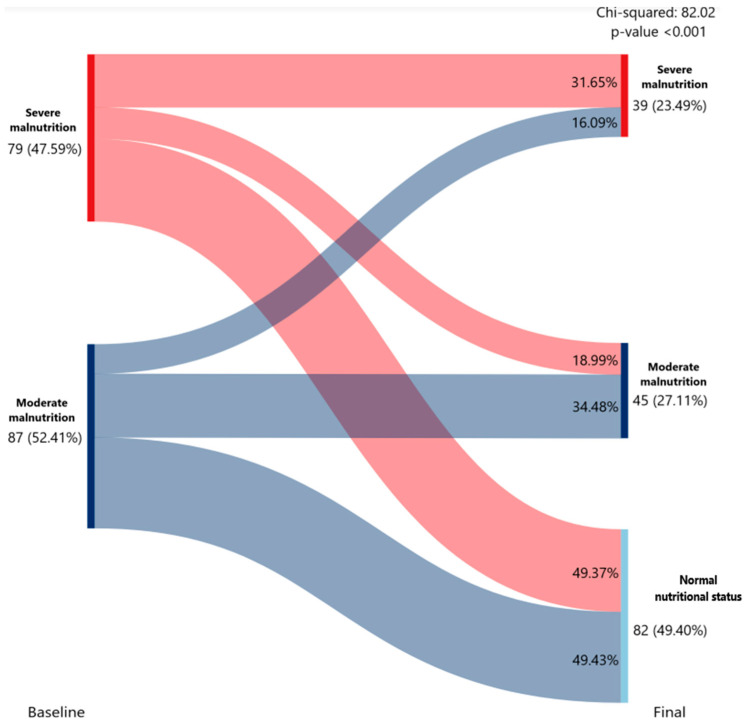
Alluvial diagram showing transitions in malnutrition status according to GLIM weight loss criteria from baseline to final assessment. This figure was created using only patients with available data at both the baseline visit and the final visit. Categories include severe malnutrition, moderate malnutrition, and absence of malnutrition. Flow width is proportional to the number of individuals transitioning between categories. Red shades denote severe malnutrition, and blue shades denote moderate malnutrition; lighter shades indicate transitions to the absence of malnutrition. The association between baseline and final malnutrition status was assessed using the chi-squared test.

Among patients with data available at both the baseline and final visits for the GLIM weight-loss criteria, 47.31% were classified as moderate malnutrition (>5% weight loss in the last six months) and severe malnutrition (>10% in the last six months) at baseline. Of those initially classified as severe, malnutrition 49.37% no longer met the weight-loss criteria at the final visit. Similarly, 48.86% of patients initially classified as moderate (52.69% of the group) also stopped meeting the GLIM weight-loss criteria, regaining normal nutritional status (Figure 4).

The percentage of patients with reduced muscle mass decreased slightly from 68.5% to 63.4%, although this change was not statistically significant (*p* = 0.1489) (Table 2). (Figure S2, Supplementary Material).

**Figure 5:**
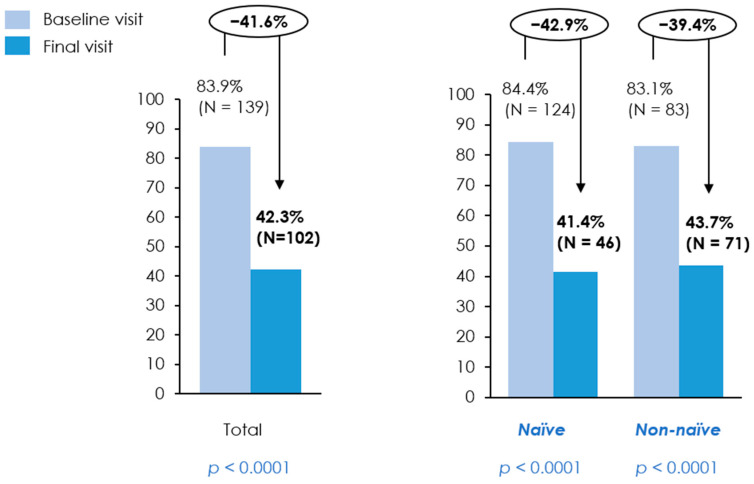
Reduced food intake according to the GLIM etiological criteria. Bar charts depict the proportion of patients meeting the GLIM criterion for reduced food intake at baseline and final visits for the total cohort, treatment-naïve patients, and non-naïve patients. Percentages and absolute numbers (N) are reported above each bar. Arrows indicate the relative percentage change between baseline and final visits. *p*-values were calculated using a *t*-test or Wilcoxon signed-rank test for continuous variables, depending on data distribution, and the McNemar test for categorical variables.

A marked improvement was observed in food intake. At baseline, 83.9% of patients reported reduced food intake, which decreased to 42.3% at follow-up (*p* < 0.0001), indicating significant nutritional recovery. A similar result was observed in both the naïve and non-naïve populations (Table 2) (Figure 5).

The proportion of patients meeting the GLIM inflammation criteria decreased substantially, from 91.7% at baseline to 81.4% at the final visit (*p* = 0.0015), indicating a significant reduction in the inflammatory burden. This improvement was observed in both cohorts, with statistically significant reductions among naïve (*p* = 0.0140) and non-naïve patients (*p* = 0.0423) (Table 2) (Figure S3, Supplementary Material).

### 3.4. Evolution of Morphofunctional Status

BIA revealed stability in most body composition parameters across visits in the overall population (all *p* > 0.05). However, among non-naïve patients, significant improvements were observed in FFMI (17.20 ± 2.49 to 17.51 ± 2.66 kg/m^2^, *p* = 0.0159) and ASMI (6.33 ± 1.19 to 6.39 ± 1.35 kg, *p* = 0.0248), indicating enhanced muscle preservation and nutritional recovery (Table 3). These changes suggest better preservation or restoration of lean body mass and skeletal muscle, which are key determinants of functionality and prognosis.

**Figure 6:**
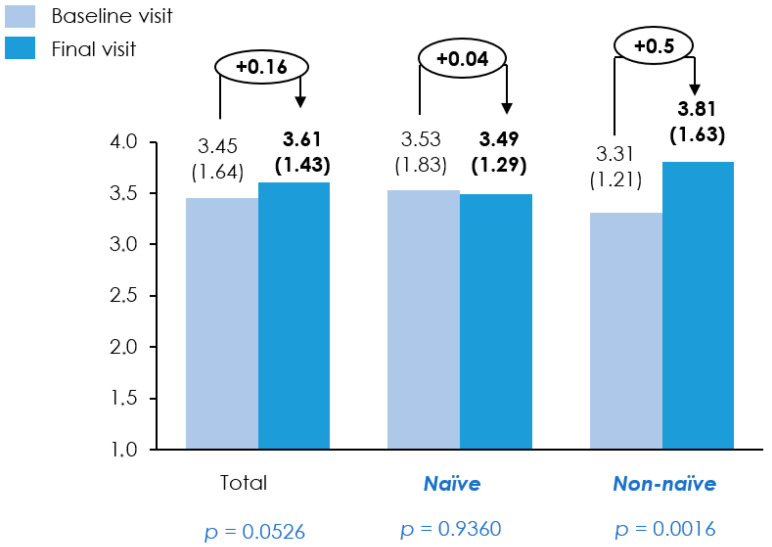
Rectus femoris cross-sectional area (RF CSA) at baseline and final visit. Bars represent mean RF CSA (SD) measured by ultrasound at baseline and final visit in the overall population, treatment-naïve patients, and non-naïve patients. Baseline and final values are shown with the absolute change between visits indicated above each group. Comparisons were performed using a *t*-test or Wilcoxon signed rank test, depending on data distribution; *p*-values are shown below each group.

Nutritional ultrasound measurements revealed that, across the entire population, most parameters exhibited slight changes between baseline and the final measurements, with no substantial differences observed. The mean RF-CSA increased from 3.45 cm^2^ to 3.61 cm^2^, approaching statistical significance (*p* = 0.0526) (Figure 6). SAT, RF-CIR, and RF-X remained largely unchanged (*p* > 0.68). For RF-Y, slight variations were observed, with relaxation and contraction measures showing small but statistically significant differences (*p* = 0.0449 and *p* = 0.0214, respectively). In the naïve subgroup, all measured parameters remained relatively stable, with no significant changes observed throughout the study period. In contrast, the non-naïve group demonstrated more pronounced changes. The RF-CSA increased significantly from 3.31 cm^2^ to 3.81 cm^2^ (*p* = 0.0016), suggesting a meaningful improvement. Also, there was a modest but statistically significant increase in RF-Y during contraction, rising from 1.60 cm to 1.70 cm (*p* = 0.0083) (Table 3).

Abdominal ultrasound measurements across the total population revealed minor decreases in T-SAT, S-SAT, and PAT from baseline to final assessment, but none were statistically significant (*p* > 0.19). In naïve patients, T-SAT and S-SAT decreased marginally, while PAT increased slightly (*p* = 0.0597), indicating a trend toward significance. Among non-naïve participants, all parameters reduced modestly; however, these changes were not significant (*p* > 0.05) (Table 3).

**Figure 7:**
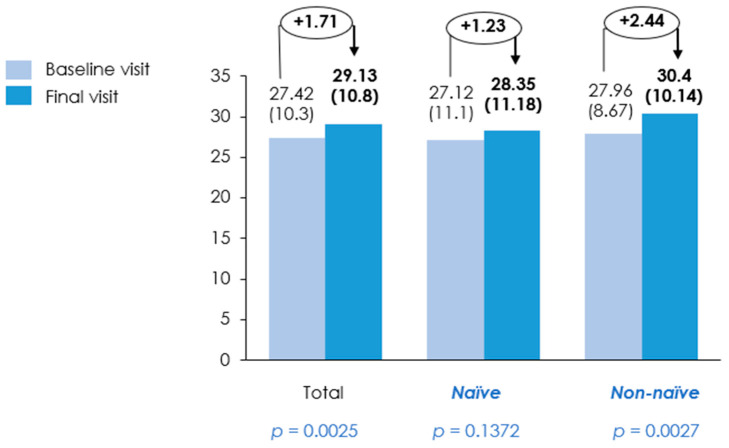
Muscle strength (kg) at baseline and final visit. Bar represents mean muscle strength with standard deviation (SD) measured at baseline and final visit in the overall population, treatment-naïve patients, and non-naïve patients. Mean values (SD) are displayed above each bar, with absolute change between visits indicated above each group. Baseline and final visits were compared using a *t*-test or Wilcoxon signed rank test for continuous variables, depending on data distribution; *p*-values are shown below each group.

**Figure 8:**
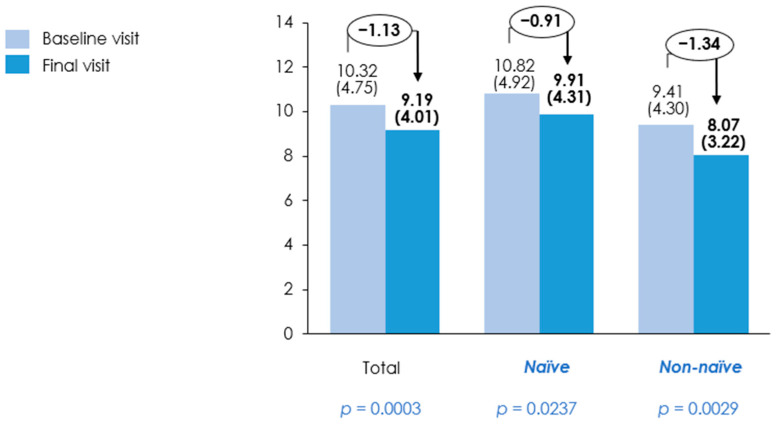
Timed Up and Go (TUG) test performance at baseline and final visit. Bars represent mean TUG time (seconds) with standard deviation (SD) measured at baseline and final visit in the overall population, treatment-naïve patients, and non-naïve patients. Mean values (SDs) are displayed above each bar, with absolute change between visits indicated above each group. Baseline and final visits were compared using a *t*-test or Wilcoxon signed rank test for continuous variables, depending on data distribution; *p*-values are shown below each group.

Muscle strength significantly improved in the overall population over the three-month period from 27.42 ± 10.28 kg to 29.13 ± 10.81 kg (*p* = 0.0025). For the naïve group, it increased from 27.12 ± 11.11 kg to 28.35 ± 11.18 kg, although this change was not statistically significant (*p* = 0.1372). In contrast, the non-naïve group showed a significant improvement from 27.96 ± 8.67 kg to 30.40 ± 10.14 kg (*p* = 0.0027) (Table 3 and Figure 7). Functional capacity, as assessed by the TUG test, exhibited a significant improvement from 10.32 ± 4.75 s to 9.19 ± 4.01 s (*p* = 0.0003) in the overall population. TUG improved significantly in both groups: the naïve group from 10.82 ± 4.92 s to 9.91 ± 4.31 s (*p* = 0.0237), and the non-naïve group from 9.41 ± 4.30 s to 8.07 ± 3.22 s (*p* = 0.0029) (Table 3 and Figure 8).

Biochemical parameters were evaluated to assess the nutritional and inflammatory status. Across the total population, albumin increased slightly from baseline to final (3.92 to 4.06 g/dL), and prealbumin rose from 21.95 to 23.85 mg/dL. Neither change was statistically significant (*p* > 0.22). CRP decreased from 3.49 to 2.66 mg/L (*p* = 0.9671), and the CRP/prealbumin ratio declined from 0.20 to 0.12 (*p* = 0.5012). In naïve participants, albumin and prealbumin showed modest increases (*p* = 0.8926 and *p* = 0.0648, respectively), CRP decreased slightly, and the CRP/prealbumin ratio fell (*p* = 0.4600). Among non-naïve participants, albumin exhibited a notable rise (3.87 to 4.10 g/dL, *p* = 0.0523), while prealbumin and CRP changes were minimal, and the CRP/prealbumin ratio remained stable (*p* = 0.9312) (Table 3).

## 4. Discussion

The present study highlighted the effectiveness of a three-month comprehensive nutritional support program—comprising a novel cHPHC-ONS, individualized dietary counseling, and structured physical exercise regimen—in improving nutritional status, functional capacity, nutritional status, and body composition in malnourished patients with cancer. Notably, this is the first investigation to assess and directly compare the impact of cHPHC-ONS between treatment-naïve and non-naïve malnourished patients with cancer, providing valuable insights into their differential responses to nutritional intervention. In this study, we opted for a multidisciplinary approach combining nutritional supplementation, individualized dietary advice, and physical exercise, as this strategy can improve patient outcomes, allow for faster recovery from malnutrition, better maintenance of muscle mass, and enhanced adherence to nutritional therapy in oncology patients [36].

Our study showed substantial nutritional recovery, with the proportion of patients meeting GLIM criteria for malnutrition decreasing from 100% at baseline to 76.2% after three months (*p* < 0.0001). Notable reductions were observed in the proportion of patients meeting GLIM weight loss criteria (>5% in six months: 46.1% to 27.5%; >10% over longer periods: 46.5% to 22.5%; *p* < 0.0001), GLIM reduced-food-intake criteria (83.9% to 42.3%; *p* < 0.0001), and GLIM inflammation criteria (91.7% to 81.4%; *p* = 0.0015). In comparison, a previous multicenter randomized trial (EFFORT trial) among hospitalized patients at nutritional risk found that 61.6% met modified GLIM criteria for malnutrition, with GLIM-positive patients experiencing higher adverse clinical outcomes (27.9% vs. 19.0%; adjusted odds ratio [OR] 1.53; 95% confidence interval [CI] 1.22–1.93, *p* < 0.001). Individualized nutritional therapy in this study significantly reduced adverse outcomes among GLIM-positive patients (31.0% vs. 25.0%, OR 0.69, 95% CI 0.53–0.9, *p* = 0.007) [42]. Additionally, reduced food intake at baseline should be interpreted in the context of impaired functional status, as patients with ECOG 3 are limited to self-care and confined to bed or chair for more than half of waking hours, a level of disability that is closely associated with malnutrition and nutritional vulnerability in patients with cancer [43,44].

The Málaga Declaration establishes a framework aligned with GLIM criteria, incorporating muscle and fat mass evaluation into a holistic nutritional assessment [45]. Our three-month nutritional support program had a positive impact on the morphofunctional characteristics of patients. BIA showed no significant changes in most body composition parameters overall or in naïve patients, which is consistent with the reports of Kang et al. [46]. However, the non-naïve group in our study demonstrated significant increases in FFMI (*p* = 0.0159) and ASMI (*p* = 0.0248), which aligns with findings from Grupińska et al., who reported significant improvements in FFMI (from 17.71 kg/m^2^ to 18.14 kg/m^2^, *p* = 0.0012) [31].

Our study also observed an increase in RF-CSA from 3.45 cm^2^ to 3.61 cm^2^, with the change approaching statistical significance (*p* = 0.0526), suggesting a potential benefit of ONS in promoting muscle mass accretion. This trend is consistent with Cornejo-Pareja et al., who reported a significant increase in muscle mass accretion (*p* < 0.05) in patients receiving a cHPHC-ONS [30]. Similarly, Vegas-Aguilar et al. demonstrated a significant improvement in RF-CSA, increasing from 3.17 cm^2^ to 3.50 cm^2^ (*p* < 0.001) [47].

A statistically significant improvement in muscle strength was observed, with muscle strength (handgrip dynamometry) increasing from 27.42 kg at baseline to 29.13 kg at follow-up (*p* = 0.0025), indicating enhanced muscular performance and improved functional status and physical resilience, which are predictive of better clinical outcomes. This aligns with findings from a single-center randomized trial in advanced patients with cancer, where a leucine-rich ONS significantly improved handgrip strength from 35.8 ± 9.8 kg to 37.6 ± 10.0 kg over three months [48]. Our results are further supported by Cornejo-Pareja et al. (2021), who reported a significant increase in handgrip strength (*p* < 0.05) in patients receiving a cHPHC-ONS [30]. Similarly, Kang et al. (2024) demonstrated a significant improvement in handgrip strength (*p* = 0.002) following an eight-week ONS regimen in patients with cancer cachexia [46]. Also, Vegas-Aguilar et al. (2025) demonstrated a significant increase in handgrip strength (from 24.3 kg to 25.6 kg, *p* = 0.001) in malnourished outpatients (with or without cancer) receiving ONS [47].

Functional capacity, assessed via the TUG test, also improved significantly, with mean completion time decreasing from 10.32 s to 9.19 s (*p* = 0.0003), reflecting enhanced mobility and physical performance. This aligns with findings from Kang et al., who reported a significant improvement in functional capacity through a four-meter walk test (*p* = 0.021) among patients with solid tumors undergoing systemic chemotherapy and receiving ONS [46], and Vegas-Aguilar et al., who observed a significant reduction in TUG time from 7.54 to 7.07 s (*p* = 0.001) in malnourished outpatients [47].

The cHPHC-ONS utilized in our study is distinguished by its content of ≥2.1 kcal/mL, 32 g of protein per 200 mL, with a combination of high-quality whey protein and casein, and around 3.6 g of intrinsic leucine per bottle. In combination with personalized dietary counseling and a structured physical exercise program, this formulation led to significant improvements in both muscle strength (handgrip dynamometry) and functional capacity (TUG test), strongly suggesting that proteins and leucine play a key role in supporting muscle growth and function. In a previous (2024) real-world study, López-Gómez et al. evaluated the same cHPHC-ONS as used in our study to monitor clinical outcomes in patients with DRM. Their findings showed an increase in mean handgrip strength from 22.12 ± 9.52 kg to 23.16 ± 9.69 kg over three months; however, this change did not reach statistical significance (*p* = 0.07). In contrast, our study demonstrated a significant improvement, with the mean handgrip strength rising from 27.42 ± 10.28 kg at baseline to 29.13 ± 10.81 kg after three months of comprehensive nutritional support (*p* = 0.0025) [35]. These results suggest that while in both studies interventions were associated with gains in muscle strength, our program produced a more robust and statistically significant improvement in handgrip dynamometry for the total population. This enhanced effect may be attributed to the inclusion of physical exercise guidance alongside cHPHC-ONS and dietary counseling in our study, whereas the study by López-Gómez et al. incorporated only cHPHC-ONS and dietary counseling.

The favorable improvements observed in muscle strength and muscle mass in our study are likely attributable to the combined effects of cHPHC-ONS supplementation, dietary counseling, and physical exercise (resistance training). Resistance exercise is a strong anabolic stimulus, while adequate protein and targeted supplementation enhance the exercise-induced muscle protein synthetic response and help counteract cancer-related catabolism [49]. Evidence from oncology patients supports this synergistic effect, with combined exercise and nutritional interventions showing greater benefits for preserving skeletal muscle mass, physical function, and patient-reported outcomes [50]. Together, these findings support a multimodal strategy and align with the integrated intervention applied in our study.

Our study found that nutritional and morphofunctional improvements were more pronounced in non-naïve patients compared with the naïve population. Nutritional improvement was more pronounced in non-naïve patients, with 28.4% achieving a normal nutritional status after three months, compared with 20.9% of naïve patients. Improvement in muscle strength was more pronounced and statistically significant in the non-naïve group (*p* = 0.0027) compared with a non-significant change in the naïve group (*p* = 0.1372). Improvements in functional capacity were also more pronounced in the non-naïve group (*p* = 0.0029) than in the naïve group (*p* = 0.0237). RF-CSA remained reduced slightly in the naïve group (3.53 cm^2^ to 3.49 cm^2^, *p* = 0.9360), while the non-naïve group showed a significant increase (3.31 cm^2^ to 3.81 cm^2^, *p* = 0.0016). These findings suggest that patients who transitioned to a novel cHPHC-ONS from a standard ONS may experience greater improvements in terms of functional capacity, nutritional status, and body composition. Importantly, to our knowledge, no previous studies have evaluated ONS outcomes using distinct patient cohorts, based on prior nutritional intervention exposure, making this a novel and noteworthy contribution of our study.

Notably, the study population largely comprised patients with advanced-stage cancer, with 31.2% at Stage III and 37.1% at Stage IV. This makes the observed improvements even more clinically meaningful, as advanced cancer stages are typically associated with severe metabolic derangements, systemic inflammation, and poor prognosis [51]. Achieving significant gains in nutritional and functional status in patients at advanced cancer stages underscores the clinical relevance and potential impact of the nutritional support program.

## 5. Study Limitations

This study was well designed to capture real-world data on the use of ONS in clinical practice, despite some inherent limitations. Firstly, the findings should be interpreted as descriptive associations reflecting temporal changes under routine clinical practice conditions, rather than as evidence of independent or causal effects of nutritional intervention. Additionally, the relatively short follow-up period of three months may not have adequately captured the long-term sustainability of the observed improvements in nutritional and functional status. Also, the absence of a control group limited the ability to attribute the observed changes exclusively to the intervention. To reduce screening bias, consecutive patient inclusion was employed, and broader inclusion criteria allowed for a more representative patient population.

Multivariable analyses were not performed, which represents a limitation of the study. Given the observational real-world design, the descriptive focus of the objectives, and the reduction in effective sample size after stratification, multivariable modeling was considered inappropriate and potentially unstable. In addition, comparisons between naïve and non-naïve patients were not adjusted for baseline characteristics, which may have influenced the observed differences between cohorts. Consequently, the results describe temporal changes in nutritional, functional, and body composition status but do not allow adjustment for potential confounding factors or assessment of independent associations.

Additionally, reliance on medical records may have resulted in missing data, potentially affecting the robustness and validity of the study’s conclusions.

## 6. Conclusions

The present study showed that a structured and comprehensive nutritional support program, combining a novel cHPHC-ONS, individualized dietary counseling, and physical activity guidance, significantly improved nutritional and morphofunctional status in malnourished patients with advanced-stage cancer. The results demonstrate a remarkable and statistically significant reduction in the prevalence of malnutrition, according to the GLIM criteria, weight loss, and inflammation. Muscle strength and functional capacity improved markedly, with statistically significant gains in handgrip strength and TUG test performance and maintenance of the phase angle as a prognostic indicator of cellular health. Notably, patients who transitioned to cHPHC-ONS after inadequate response to initial ONS (non-naïve group) experienced greater benefits than the naïve group across most outcomes. These findings establish the effectiveness of a novel cHPHC-ONS and support the integration of structured, personalized nutritional and physical exercise strategies into oncology care.

## Figures and Tables

**Table 1:** Baseline demographic and clinical characteristics, GLIM criteria, bioelectrical impedance and functional parameters.

Variable	Total (N = 230)	Naïve (N = 147)	Non-Naïve (N = 83)	*p*-Value * (Naïve vs. Non-Naïve)
Anthropometric and demographic variables				
Age (SD), years (mean, SD)	64.38 (11.11)	64.15 (11.36)	64.79 (10.71)	0.8087
Sex				
Female	84 (40.2%)	59 (44.03%)	25 (33.33%)	0.1491
Male	125 (59.8%)	75 (55.97%)	50 (66.67%)	
Body mass index, kg/m^2^ (mean, SD)	23.25 (4.35)	25.59 (4.31)	22.66 (4.39)	0.1216
Clinical variables				
Type of diagnosed cancer, n (%)				
Head and neck cancer	84 (36.7%)	51 (34.9%)	33 (39.8%)	0.4436
Gastric cancer	35 (15.3%)	22 (15.1%)	13 (15.7%)	0.8877
Colon and rectal cancer	32 (14.0%)	18 (12.3%)	14 (16.9%)	0.3306
Lung cancer	30 (13.1%)	20 (13.7%)	10 (12.1%)	0.7363
Cancer stage at diagnosis, n (%)				
I	20 (9.1%)	12 (8.6%)	8 (9.9%)	0.7029
II	50 (22.6%)	32 (22.9%)	18 (22.2%)	0.9885
III	69 (31.2%)	41 (29.3%)	28 (34.6%)	0.353
IV	82 (37.1%)	55 (39.3%)	27 (33.3%)	0.4576
ECOG status:				
0	71 (30.9%)	46 (31.3%)	25 (30.1%)	0.8534
1	108 (47.0%)	62 (42.2%)	46 (55.4%)	0.0532
2	45 (19.6%)	35 (23.8%)	10 (12.1%)	0.0308
3	6 (2.6%)	4 (2.7%)	2 (2.4%)	0.8868
Comorbidities:				
Chronic lung disease	19 (8.3%)	14 (9.5%)	5 (6.0%)	0.3545
Cerebrovascular disease	12 (5.2%)	6 (4.1%)	6 (7.2%)	0.3026
Chronic heart failure	11 (4.8%)	8 (5.4%)	3 (3.6%)	0.7501
Uncomplicated diabetes mellitus	9 (3.9%)	3 (2.0%)	6 (7.2)	0.0744
GLIM criteria met **, n (%)	230 (100.0%)	147 (100.0%)	83 (100.0%)	
Phenotypic criteria				
Weight loss, n (%)				
>5% in the last 6 months	106 (46.1%)	72 (49.0%)	34 (41.0%)	0.2415
>10% in the last 6 months	107 (46.5%)	67 (45.6%)	40 (48.2%)	0.7026
Low BMI, n (%)				
<20 kg/m^2^ if <70 years	36 (15.7%)	20 (13.6%)	16 (19.3%)	0.2556
<22 kg/m^2^ if >70 years	22 (9.6%)	13 (8.8%)	9 (10.8%)	0.6204
Reduced muscle mass, n (%) ***	139 (68.5%)	93 (71.0%)	46 (63.9%)	0.2973
Etiological criteria				
Inflammation, n (%)	209 (91.7%)	132 (91.0%)	77 (92.8%)	0.648
Reduced food intake, n (%)	193 (83.9%)	124 (84.4%)	69 (83.1%)	0.8087
Bioelectrical impedance (mean, SD)				
PA (°),	4.82 (0.88)	4.76 (0.86)	4.85 (0.89)	0.4943
SPA (°)	0.37 (1.23)	0.55 (1.17)	0.27 (1.27)	0.168
BCM (kg)	22.88 (6.16)	22.43 (5.63)	23.15 (6.46)	0.4733
FMI, (kg/m^2^)	5.69 (3.07)	5.23 (3.30)	5.95 (2.91)	0.142
FFMI (kg/m^2^)	17.45 (2.33)	17.20 (2.49)	17.59 (2.23)	0.3021
ASMI (kg)	6.42 (1.12)	6.33 (1.19)	6.47 (1.08)	0.4215
Nutritional ultrasound (mean, SD)				
RF-CSA (cm^2^)	3.45 (1.64)	3.53 (1.83)	3.31 (1.21)	0.6061
RF-CIR (cm)	8.89 (1.68)	8.97 (1.87)	8.76 (1.29)	0.852
RF-X-axis (cm)	4.29 (4.06)	4.41 (4.39)	4.11 (3.45)	0.4945
RF-Y-axis (cm)	1.59 (2.04)	1.78 (2.28)	1.31 (1.56)	0.1127
Contracted rectus femoris-Y axis (CF-Y-axis, cm)	1.84 (2.18)	2.01 (2.60)	1.60 (1.31)	0.4492
L-SAT (cm)	0.87 (0.79)	0.98 (0.86)	0.68 (0.60)	0.0109
Abdominal ultrasound (mean, SD)				
T-SAT (cm)	1.67 (1.50)	1.71 (1.44)	1.60 (1.62)	0.6525
S-SAT (cm)	0.81 (0.84)	0.83 (0.74)	0.77 (1.00)	0.7018
PAT (cm)	0.63 (0.57)	0.61 (0.50)	0.67 (0.70)	0.5525
Functional parameters				
Handgrip strength (kg)	27.42 (10.28)	27.12 (11.11)	27.96 (8.67)	0.5287
TUG (s)	10.32 (4.75)	10.82 (4.92)	9.41 (4.30)	0.0438
Biochemical parameters (mean, SD)				
Albumin (g/dL)	3.92 (0.54)	3.95 (0.51)	3.87 (0.58)	0.335
Prealbumin (mg/dL)	21.95 (6.73)	21.46 (6.93)	22.78 (6.35)	0.2363
CRP (mg/L)	3.49 (5.95)	3.32 (5.42)	3.76 (6.72)	0.5431
CRP/prealbumin ratio	0.20 (0.48)	0.18 (0.38)	0.22 (0.62)	0.5166

Abbreviations: ASMI, appendicular skeletal muscle index; BCM, body cell mass; CRP, C-reactive protein; ECOG, Eastern Cooperative Oncology Group; FFMI, fat-free mass index; FMI, fat mass index; L-SAT, subcutaneous adipose fat of leg, PA, phase angle; PAT, preperitoneal adipose tissue; RF-CSA, rectus femoris cross-sectional area; RF-CIR, circumference of quadriceps rectus femoris; RF-X-axis, rectus femoris—X axis; RF-Y-axis, rectus femoris—Y axis; CF-Y-axis, contracted rectus femoris—Y axis; SPA, standardized phase angle; S-SAT, superficial subcutaneous adipose tissue; T-SAT, total subcutaneous adipose tissue. SD, Standard deviation. * *p*-values are obtained from the *t*-test or the Wilcoxon signed-rank test for continuous variables, depending on their distribution, and the McNemar test for categorical variables. ** To meet the GLIM criteria, at least one phenotypic criterion AND at least one etiological criterion must be met. *** Reduced muscle mass according to GLIM criteria recommendations (men ≤ 17 kg/m^2^, women < 15 kg/m^2^). NOTE: For each time point, mean (SD), and percentage were calculated using patients for whom data were available (baseline and final visit, respectively).

**Table 2:** Evolution of nutritional status in enrolled patients (GLIM criteria).

	Total (N = 230)			Naïve (N = 147)			Non-Naïve (N = 83)		
Variable	Baseline Visit	Final Visit	*p* *	Baseline Visit	Final Visit	*p* *	Baseline Visit	Final Visit	*p* *
Fulfillment of GLIM criteria **, n (%)	230 (100.0%) (N = 230)	131 (76.2%) (N = 182)	<0.0001	147 (100.0%) (N = 147)	83 (79.1%) (N = 111)	<0.0001	83 (100.0%) (N = 83)	48 (71.6%) (N = 71)	<0.0001
Phenotypic criteria									
Weight loss GLIM criteria, n (%)									
>5% in the last 6 months	106 (46.1%)	50 (27.5%)	<0.0001	72 (49.0%)	33 (29.7%)	<0.0001	34 (41.0%)	17 (23.9%)	<0.0001
>10% in the last 6 months	107 (46.5%)	41 (22.5%)		67 (45.6%)	29 (26.1%)		40 (48.2%)	12 (16.9%)	
Low BMI GLIM criteria, n (%)									
<20 kg/m^2^ if <70 years	36 (15.7%)	29 (15.9%)	0.9109	20 (13.6%)	16 (14.4%)	0.9486	16 (19.3%)	13 (18.3%)	0.7213
<22 kg/m^2^ if ≥70 years	22 (9.6%)	16 (8.8%)		13 (8.8%)	9 (8.1%)		9 (10.8%)	7 (9.9%)	
Reduced muscle mass, n (%)	139 (68.5%)	102 (63.4%)	0.1489	93 (71.0%)	62 (64.6%)	0.1446	46 (63.9%)	40 (61.5%)	0.6957
Etiological criteria									
Reduced food intake GLIM criteria, n (%)	193 (83.9%)	77 (42.3%)	<0.0001	124 (84.4%)	46 (41.4%)	<0.0001	69 (83.1%)	31 (43.7%)	<0.0001
Inflammation GLIM criteria, n (%)	209 (91.7%)	144 (81.4%)	0.0015	132 (91.0%)	87 (80.6%)	0.0140	77 (92.8%)	57 (82.6%)	0.0423

GLIM, Global Leadership Initiative on Malnutrition; BMI, body mass index. * *p*-values are obtained from the *t*-test or the Wilcoxon signed-rank test for continuous variables, depending on their distribution, and the McNemar test for categorical variables. ** To meet the GLIM criteria, at least one phenotypic criterion AND at least one etiological criterion must be met. NOTE: For each time point, percentages were calculated using, as a denominator, only those patients for whom data were available (baseline and final visit, respectively).

**Table 3:** Evolution of morphofunctional parameters of enrolled patients.

Variable	Total (N = 230)			Naïve (N = 147)			Non-Naïve (N = 83)		
	Baseline	Final	*p*-Value	Baseline	Final	*p*-Value	Baseline	Final	*p*-Value *
Bioelectrical impedance (mean, SD)									
PA (°)	4.82 (0.88)	4.82 (0.83)	0.2092	4.85 (0.89)	4.79 (0.88)	0.0751	4.76 (0.86)	4.86 (0.75)	0.7801
SPA (°)	0.37 (1.23)	0.30 (1.27)	0.6502	0.27 (1.27)	0.20 (1.26)	0.6019	0.55 (1.17)	0.44 (1.30)	0.9732
BCM (kg)	22.88 (6.16)	22.75 (6.11)	0.9604	23.15 (6.46)	22.72 (6.33)	0.5282	22.43 (5.63)	22.81 (5.83)	0.3021
FMI (kg/m^2^)	5.69 (3.07)	5.59 (2.70)	0.1439	5.95 (2.91)	5.84 (2.63)	0.1286	5.23 (3.30)	5.19 (2.79)	0.8342
FFMI (kg/m^2^)	17.45 (2.33)	17.53 (2.41)	0.2169	17.59 (2.23)	17.54 (2.25)	0.6627	17.20 (2.49)	17.51 (2.66)	0.0159
ASMI (kg)	6.42 (1.12)	6.40 (1.19)	0.5811	6.47 (1.08)	6.42 (1.07)	0.4416	6.33 (1.19)	6.39 (1.35)	0.0248
Nutritional ultrasound (mean, SD)									
RF-CSA (cm^2^)	3.45 (1.64)	3.61 (1.43)	0.0526	3.53 (1.83)	3.49 (1.29)	0.9360	3.31 (1.21)	3.81 (1.63)	0.0016
RF-CIR (cm)	8.89 (1.68)	8.91 (1.14)	0.8571	8.97 (1.87)	8.85 (1.09)	0.7350	8.76 (1.29)	9.00 (1.22)	0.1017
RF-X-axis (cm)	4.29 (4.06)	4.12 (4.19)	0.6833	4.41 (4.39)	4.27 (4.85)	0.3196	4.11 (3.45)	3.86 (2.85)	0.5595
RF-Y-axis (cm)	1.59 (2.04)	1.65 (2.84)	0.0449	1.78 (2.28)	1.88 (3.57)	0.9964	1.31 (1.56)	1.30 (0.77)	0.0008
Contracted rectus femoris—Y axis (CF-Y-axis, cm)	1.84 (2.18)	2.05 (3.25)	0.0214	2.01 (2.60)	2.29 (4.09)	0.4769	1.60 (1.31)	1.70 (1.24)	0.0083
L-SAT (cm)	0.87 (0.79)	0.87 (0.74)	0.6985	0.98 (0.86)	0.94 (0.70)	0.4806	0.68 (0.60)	0.76 (0.80)	0.6343
Abdominal ultrasound (mean, SD)									
T-SAT (cm)	1.67 (1.50)	1.57 (1.25)	0.7599	1.71 (1.44)	1.63 (1.27)	0.5002	1.60 (1.62)	1.46 (1.23)	0.7363
S-SAT (cm)	0.81 (0.84)	0.75 (0.67)	0.9363	0.83 (0.74)	0.80 (0.70)	0.5283	0.77 (1.00)	0.67 (0.62)	0.5493
PAT (cm)	0.63 (0.57)	0.61 (0.59)	0.1926	0.61 (0.50)	0.67 (0.64)	0.0597	0.67 (0.70)	0.49 (0.47)	0.7091
Functional parameters (mean, SD)									
Handgrip strength (kg)	27.42 (10.3)	29.13 (10.8)	0.0025	27.12 (11.1)	28.35(11.18)	0.1372	27.96 (8.67)	30.4 (10.14)	0.0027
TUG (s)	10.32 (4.75)	9.19 (4.01)	0.0003	10.82 (4.92)	9.91 (4.31)	0.0237	9.41 (4.30)	8.07 (3.22)	0.0029
Biochemical parameters (mean, SD)									
Albumin (g/dL)	3.92 (0.54)	4.06 (0.47)	0.4277	3.95 (0.51)	4.04 (0.52)	0.8926	3.87 (0.58)	4.10 (0.37)	0.0523
Prealbumin (mg/dL)	21.95 (6.73)	23.85 (6.61)	0.2232	21.46 (6.93)	23.83 (6.96)	0.0648	22.78 (6.35)	23.88 (6.12)	0.5494
CRP (mg/L)E	3.49 (5.95)	2.66 (3.91)	0.9671	3.32 (5.42)	2.52 (3.60)	0.8462	3.76 (6.72)	2.86 (4.36)	0.6624
CPR/prealbumin ratio	0.20 (0.48)	0.12 (0.20)	0.5012	0.18 (0.38)	0.12 (0.19)	0.4600	0.22 (0.62)	0.13 (0.21)	0.9312

Abbreviations: ASMI, appendicular skeletal muscle index; BCM, body cell mass; CRP, C-reactive protein; FFMI, fat-free mass index; FMI, fat mass index; PA, phase angle; L-SAT: subcutaneous adipose fat of leg; PAT, preperitoneal adipose tissue; RF-CSA, rectus femoris cross-sectional area; RF-CIR, circumference of quadriceps rectus femoris; RF-X-axis, rectus femoris—X axis; RF-Y-axis, CF-Y-axis, contracted rectus femoris—Y axis; S-SAT, superficial subcutaneous adipose tissue; SAT, subcutaneous adipose tissue; SPA, standardized phase angle; T-SAT, total subcutaneous adipose tissue; TUG, test Timed Up and Go; CRP, C-reactive protein. * *p*-values are obtained from a *t*-test or the Wilcoxon signed-rank test for continuous variables, depending on their distribution, as well as the McNemar test. NOTE: For each time point, percentages were calculated using as denominator only those patients for whom data were available (baseline and final visit, respectively).

## Data Availability

The datasets generated and/or analyzed during the current study are available from the corresponding author upon request.

## Supplementary Materials

The following supporting information can be downloaded at: https://www.mdpi.com/article/10.3390/nu18091398/s1. Supplementary Material File S1: Table S1: Nutritional Composition per serving (200 mL) of the concentrated high-protein, high-calorie oral nutrition supplement used (Meritene^®^ Clinical Extra Protein). Figure S1: Weight loss (Phenotypic GLIM Criteria). Figure S2: Reduced muscle mass (Phenotypic GLIM Criteria). Figure S3: Inflammation (Etiological GLIM Criteria). Supplementary Material File S2. Participant sites collaborators. Supplementary Material File S3. Dietary recommendations and physicial exercise v2.0.

## Abbreviations

The following abbreviations are used in this manuscript:

ONS	Oral Nutritional Supplement
cHPHC-ONS	Concentrated High-Protein, High-Calorie Oral Nutritional Supplement
GLIM	Global Leadership Initiative on Malnutrition
BIA	Bioelectrical Impedance Analysis
TUG	Timed Up and Go (test)
FFMI	Fat-Free Mass Index
ASMI	Appendicular Skeletal Muscle Index
RF-CSA	Rectus Femoris Cross-Sectional Area
RF-CIR	Rectus Femoris Circumference
RF-X	Rectus Femoris Horizontal Axis
RF-Y	Rectus Femoris Vertical Axis
L-SAT	Subcutaneous Adipose Tissue of Leg
T-SAT	Total Subcutaneous Adipose Tissue
S-SAT	Superficial Subcutaneous Adipose Tissue
PAT	Preperitoneal Adipose Tissue
SPA	Standardized Phase Angle
PA	Phase Angle
BCM	Body Cell Mass
FMI	Fat Mass Index
CF-Y-axis	Contracted Rectus Femoris—Y Axis
PRO	Patient-Reported Outcome
eCRF	Electronic Case Report Form
SD	Standard Deviation
N	Number (Sample Size)
ECOG	Eastern Cooperative Oncology Group
IRB	Institutional Review Board
CEICA	Comité de Ética de la Investigación de la Comunidad Autónoma de Aragón
GPEP	Good Pharmacoepidemiological Practice
ISPE	International Society for Pharmacoepidemiology
DRM	Disease-Related Malnutrition
ESPEN	European Society for Clinical Nutrition and Metabolism
