# Supplementary material for: Effectiveness of a Comprehensive Program Including a Novel Concentrated High-Protein, High-Calorie Oral Nutritional Supplement to Enhance Nutritional and Morphofunctional Recovery in Malnourished Patients with Cancer: The ONAVIDA Study
Source: Nutrients. 2026 Apr 29;18(9):1398. doi: 10.3390/nu18091398 (PMC13165281; doi:10.3390/nu18091398)
Supplement: Supplementary file 1 [file nutrients-18-01398-s001.zip › Supplementary material File S1.pdf]

Supplementary Materials

Table S1 Nutritional Composition per serving (200mL) of the concentrated high-protein, high-calorie Oral Nutrition Supplement used (Meritene® Clinical Extra Protein).

**Nutritional component (Unit)**	**One bottle of 200 mL**
**Energy (kcal / kJ)**	420/1760
**Proteins (g)**	32 (50% Whey protein/50% Casein)
L-Leucine (g)	3.6
**Fats (g)**	21
Saturated (g)	2.2
Monounsaturated (g)	12
Polyunsaturated (g)	5.2
**Carbohydrates (g)**	26
Sugars (g)	15
Lactose (g)	1

**Nutritional component (Unit)**	**One bottle of 200 mL**
**Vitamins**	
Vitamin A (mcg)	240
Vitamin D (mcg)	4
Vitamin E (mg)	4
Vitamin K (mcg)	44
Vitamin C (mg)	46
Thiamin (mg)	1.3
Riboflavin (mg)	1
Vitamin B6 (mg)	0.6
Niacin (mg/mg NE)	1
Folic Acid (mg)	108
Vitamin B12 (mg)	2.4
Pantothenic acid (mg)	1.4

**Nutritional component (Unit)**	**One bottle of 200 mL**
Biotin (mg)	10
**Minerals**	
Sodium (mg)	300
Potassium (mg)	460
Chloride (mg)	260
Calcium (mg)	400
Phosphorus (mg)	330
Magnesium (mg)	60
Iron (mg)	3.4
Zinc (mg)	3.6
Manganese (mg)	0.38
Copper (mg)	0.60
Iodine (mcg)	44

**Nutritional component (Unit)**	**One bottle of 200 mL**
Selenium (mcg)	20
Chromium (mcg)	18
Molybdenum (mcg)	40

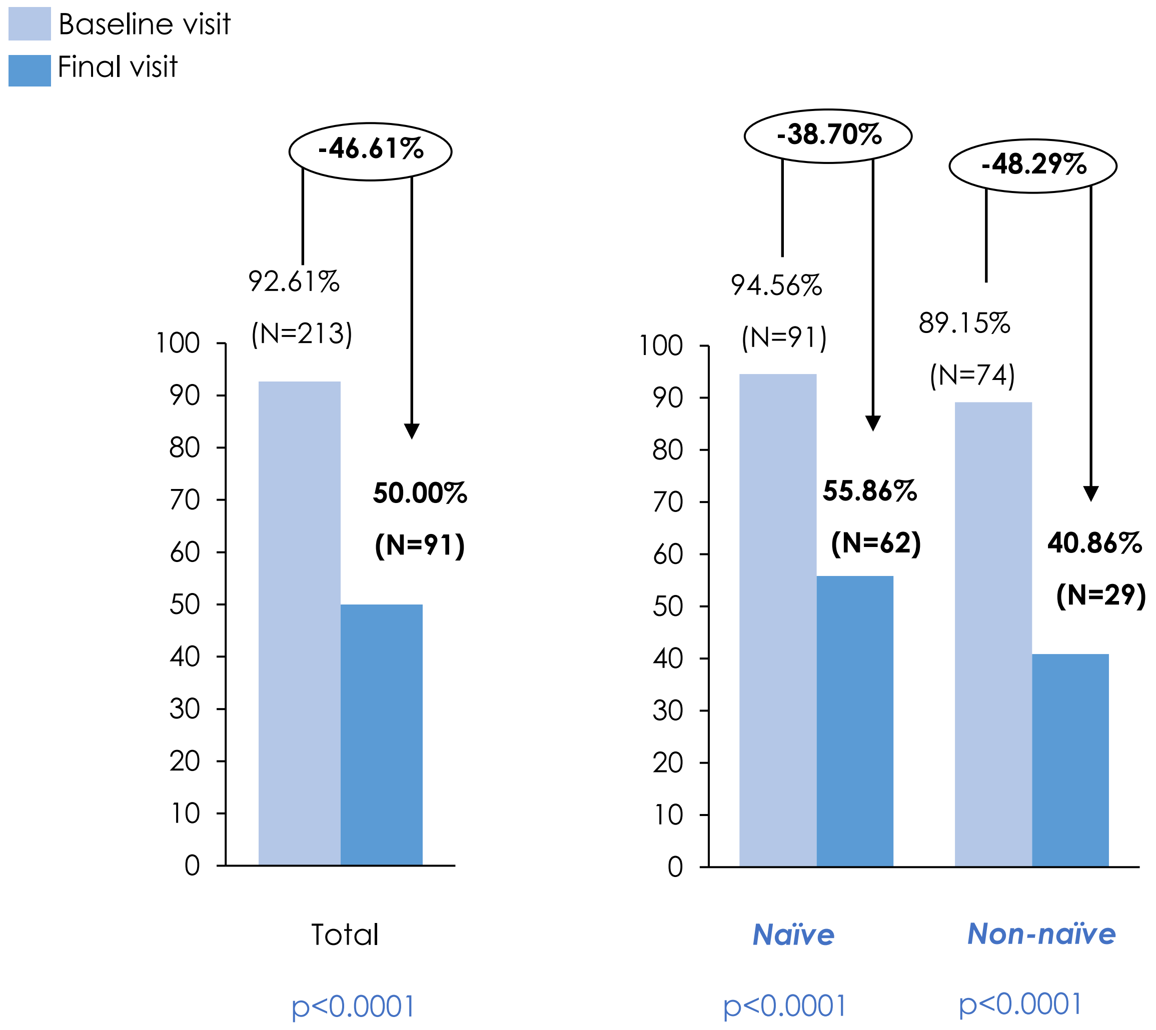

Figure. S1 Weight loss (Phenotypic GLIM Criteria). P-values are obtained from the t- test or the Wilcoxon signed-rank test for continuous variables, depending on their distribution, and the McNemar test for categorical variables. *Note: The overall proportion of patients experiencing weight loss (92.1%) is derived from two categories: those with >5% weight reduction in the past 6 months (46.1%) and those with >10% weight reduction within the same period (46.5%).*

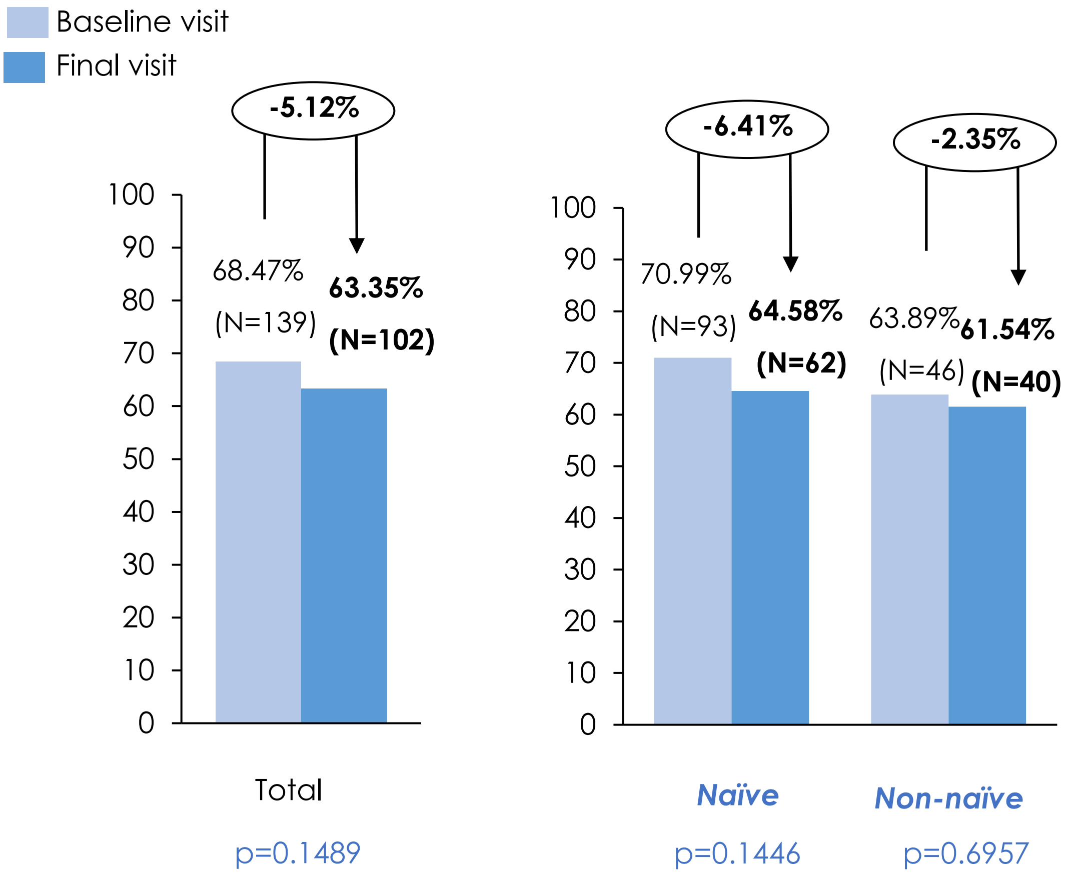

Figure. S2 Reduced muscle mass according to the GLIM phenotypic criterion. Bar charts show the proportion of patients meeting the GLIM criterion for reduced muscle mass at baseline and final visits for the total population, treatment-naïve patients, and non-naïve patients. Percentages and absolute numbers (N) are shown above each bar. Arrows indicate the relative percentage change between baseline and final visits. P values were calculated using the t test or Wilcoxon signed-rank test for continuous variables, depending on data distribution, and the McNemar test for categorical variables.

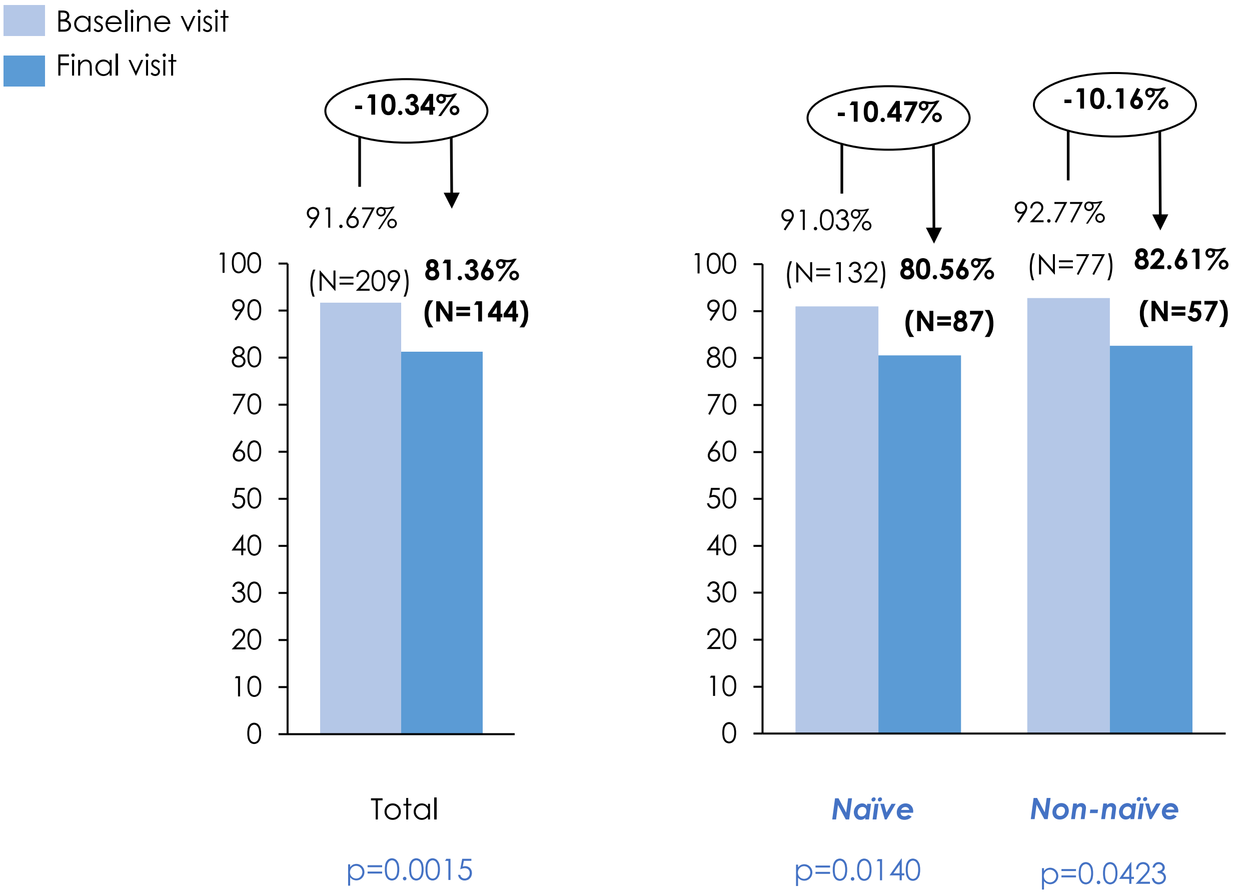

Figure. S3 Inflammation according to the GLIM etiological criterion. Bar charts show the proportion of patients meeting the GLIM criterion for inflammation at baseline and final visits for the total cohort, treatment-naïve patients, and non-naïve patients. Percentages and absolute numbers (N) are presented above each bar. Arrows indicate the relative percentage change between baseline and final visits. P values were calculated using the t test or Wilcoxon signed-rank test for continuous variables, depending on data distribution, and the McNemar test for categorical variables.
