# Supplementary material for: Effectiveness of a Comprehensive Program Including a Novel Concentrated High-Protein, High-Calorie Oral Nutritional Supplement to Enhance Nutritional and Morphofunctional Recovery in Malnourished Patients with Cancer: The ONAVIDA Study
Source: Nutrients. 2026 Apr 29;18(9):1398. doi: 10.3390/nu18091398 (PMC13165281; doi:10.3390/nu18091398)
Supplement: Supplementary file 1 [file nutrients-18-01398-s001.zip › Supplementary material File S2. Participant sites collaborators.pdf]

File S2. Participant sites collaborators

Site	Principal Investigator (PI)	Collaborators
Virgen de la Victoria University Hospital (Málaga)	José Manuel García Almeida	Rocío Fernández<br>Isabel María Vegas<br>Dr Patricia Guirado<br>Natalia Moreno
Navarra University Hospital (Pamplona)	Dr Ana Hernández-Moreno	Dr Amelia Marí Sanchis<br>Dr Ana Zugasti Murillo
Regional University Hospital of Málaga (Málaga)	Dr Gabriel Olveira	Mª García Olivares<br>Dr Nuria Porras Pérez<br>Dr Marina Barranco<br>Dr Montserrat Gonzalo Marín<br>Dr Francisco José Sánchez Torralbo<br>Dr Mª Jose Tapia Guerrero<br>Dr Mª del Carmen Bautista Recio
Torrecárdenas University Hospital (Almería)	Dr Mercedes Vázquez-Gutiérrez	Dr Mª José Jiménez Jiménez<br>Dr Josefa Teodosia Muñoz
Jiménez Díaz Foundation Hospital (Madrid)	Dr Carolina Dassen	Dr. Marta Crespo Yanguas<br>Dr. Cristina Calderón Sánchez

Site	Principal Investigator (PI)	Collaborators
Virgen del Rocío University Hospital (Seville)	Dr Pedro Pablo García - Luna	Silvia García Rey<br>Mª Carmen Roque Cuéllar<br>Ana Mª Villarubia<br>Dr Antonio Jesús Martínez Ortega<br>Irene González - Navarro<br>Rocío Vázquez
San Cecilio Clinical University Hospital (Granada)	Dr Amalia González-Jiménez	Dr Maria Luisa Fernández Soto
Son Espases University Hospital (Mallorca)	Dr Josefina Olivares-Alcolea	No aplica
León University Healthcare Complex (León)	Dr. María García-Duque	Dr. Diana Guadalupe Ariadel Cobo
Jaén University Hospital (Jaén)	Mª José Martínez-Ramírez	Dr Juan de Dios Barranco<br>Dr Elena Vera<br>Dr Ana Segarra<br>Dr Macarena Moreno
Virgen de las Nieves University Hospital (Granada)	Dr Juan Manuel-Guardia	Dr María Novo Rodríguez<br>Dr Jose M. Romero Márquez

Site	Principal Investigator (PI)	Collaborators
Juan Ramón Jiménez University Hospital (Huelva)	Dr Isabel Rebollo	Dr Luna Ojeda<br>Dr María Lainez López<br>Josefa García Alfonso<br>Antonio Fernández Toscano
Valencia Clinical University Hospital (Valencia)	Dr Miguel Civera	Dr Blanca Alabadi<br>Sandra Amores Alandí
Guadalajara University Hospital (Guadalajara)	Dr Visitación Álvarez-de Frutos	Dr Silvia Lallena
Costa del Sol Hospital (Málaga)	Dr Vicente Faus	Dr Begoña Tortajada Goitia<br>Dr Elena Álvaro Sanz<br>Iara Cacace
Cabueñes University Hospital (Gijón)	Dr Lucía Díaz-Naya	No aplica
Albacete General Hospital (Albacete)	Jose-Joaquín Alfaro Martínez	Dr Pedro Pinés Corrales<br>Dr Rosa Quílez Toboso

Site	Principal Investigator (PI)	Collaborators
		Dr Lourdes García Blasco<br>Dr César Gonzalvo Díaz
Miguel Servet University Hospital (Zaragoza)	Dr Alejandro Sanz París	Dr Beatriz Lardies
