# Supplementary material for: Effectiveness of a Comprehensive Program Including a Novel Concentrated High-Protein, High-Calorie Oral Nutritional Supplement to Enhance Nutritional and Morphofunctional Recovery in Malnourished Patients with Cancer: The ONAVIDA Study
Source: Nutrients. 2026 Apr 29;18(9):1398. doi: 10.3390/nu18091398 (PMC13165281; doi:10.3390/nu18091398)

File S3. Dietary recommendations and physical exercise regimen

General dietary recommendations

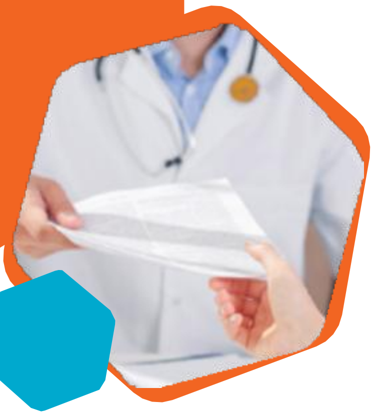

- Make sure the dishes are appetizing and have attractive colors and presentations.
- Divide the daily intake into 5-6 meals, respecting the patient's preferred meal times.
- Eat slowly and, if possible, eat food at room temperature.
- Small volumes with high nutritional content and easy digestion are better tolerated.
- Enrich the dishes to make them complete and nutritious.
- Cook food simply, preferably by baking, steaming, boiling, grilling… and avoid breaded foods and stews with lots of fat.
- Drink a minimum of 2 liters of fluid per day (water, infusions,...), preferably between meals and at room temperature.
- Consume 5 servings of fresh fruit (citrus and non-citrus) or vegetables daily.
- Eat lean or white meats (chicken, lean beef, rabbit) and fish.
- Promote a calm and pleasant atmosphere for meals, always respecting the patient's feeling of fullness or tiredness (never insist or force them to finish the meal).
- After eating, rest reclining or semi-reclining, never lying down.
- Maintain good oral hygiene; clean your teeth with a soft brush and rinse your mouth before and after each meal with chamomile or thyme infusions.

- Reduce your consumption of:
  - Animal fats
  - Foods high in added sugars and salt
  - Stimulating drinks, coffee, tea, cola...
- Avoid:
  - Alcohol consumption
  - Spices and hot peppers
  - Unpleasant smells

In situations where food intake is altered due to complications depending on the treatments they are undergoing, it will be necessary to provide specific dietary recommendations

Dietary recommendations in cases of anorexia

(lack of appetite)

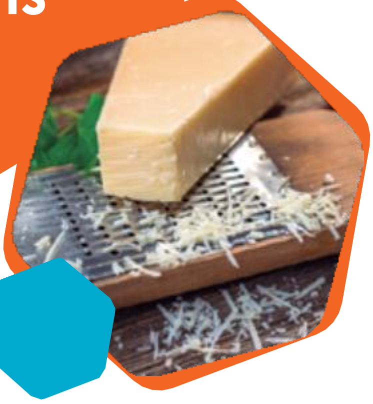

- Eat small, frequent meals.
- Adapt the texture to the patient's needs to prevent fatigue.
- Include foods of different textures and colors to make mealtimes more attractive and appetizing.
- Plan your daily menu in advance and vary your diet as much as possible.
- Take advantage of moments of peak appetite to eat foods with the highest energy and protein content: whole dairy products, pasta, rice, nuts, canned fruit...
- Nutritionally enrich dishes and drinks by adding:
    - to milk: powdered milk, fruit in syrup, honey….
    - To soups and purees: grated cheese, hard-boiled egg, ham, chopped chicken, legumes...
    - To vegetables, rice and pasta: cooked egg white, nuts, mayonnaise, tuna flakes...
- Prepare a single dish that includes both the first and second courses.
- Eat slowly and chew your food properly.
- Use foods with little aroma and at room temperature.
- Drink between main meals to avoid feeling full.
- It can help stimulate appetite:
    - Acidic fruits
    - Walk for a few minutes before meals.
- If you wake up during the night, take the opportunity to drink nutritious fluids. (milk, juices, smoothies, liquid yogurt...).

!

- Avoid:
    - Consuming skimmed, low-calorie foods
    - Liquids in meals
    - Very heavy foods or foods that may produce flatulence

EXHIBIT:Feeding patterns

Nutritional intervention is fundamental in the early stages of the disease, when the patient still presents with pre-cachexia and the metabolic alterations that lead to cachexia are still susceptible to responding to nutritional treatment.[76]

Traditional and basic adapted diets are presented as the base of the pyramid, while the use of artificial nutrition will depend on whether or not the requirements are met by said diet, and will differentiate between oral supplements, enteral or parenteral nutrition (see FIGURE 1).

In recent years, the effects of dietary patterns on the risk of cancer (and other diseases) have gained more importance, as opposed to the effects of individual nutrients.

In general, dietary patterns that show the most health benefits are based primarily on plant-based foods (including non-starchy vegetables, whole fruits, whole grains, pulses, and nuts/seeds), healthy protein sources (higher in pulses and/or fish and/or poultry, and lower in processed and red meats), and include unsaturated fats (such as monounsaturated and polyunsaturated fats).These patterns are also lower in added sugar, saturated and/or trans fats, and excess calories [77].The FIGURE 2 sample shows the recommended proportions of ca- provides macronutrients to cancer patients.

How do we know how to write a cycle?

With so-called dietary enhancers, foods that, when added to dishes, improve their caloric and protein nutritional characteristics.The most frequently used ones are:

- SPECIAL MILK: used as a beverage and in cooking.Add the powdered milk to the milk (10 tablespoons of powdered milk per liter of milk). It can be used in the preparation of smoothies (with fresh or canned fruit), desserts, soups and purees (adding a tablespoon of powdered milk), with cereals, with cocoa powder or fruit syrups, and also by adding béchamel sauce to dishes such as vegetables, pasta or potatoes.

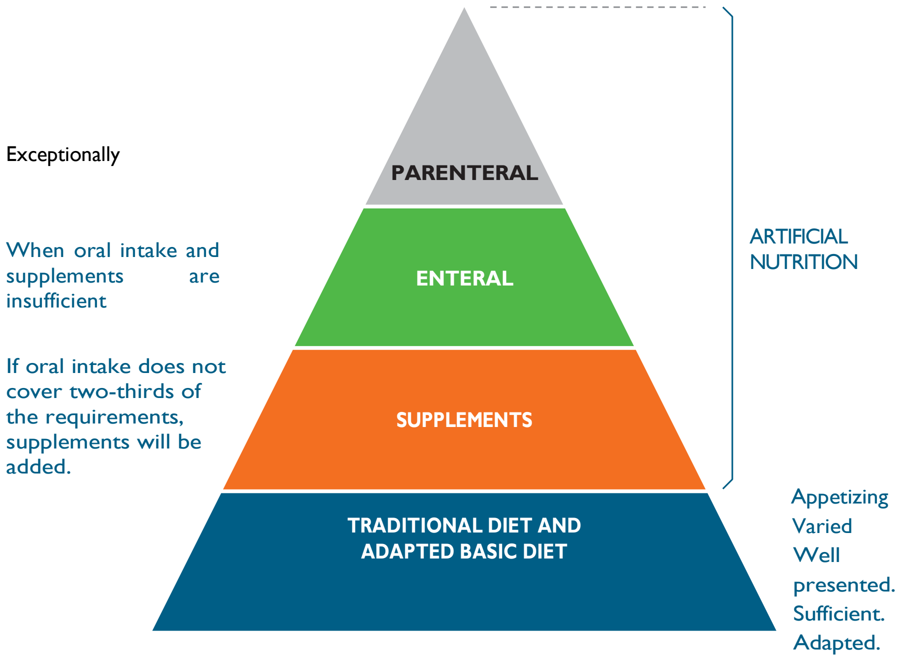

FIGURE 1 Human nutrition pyramid.

Source: own elaboration.

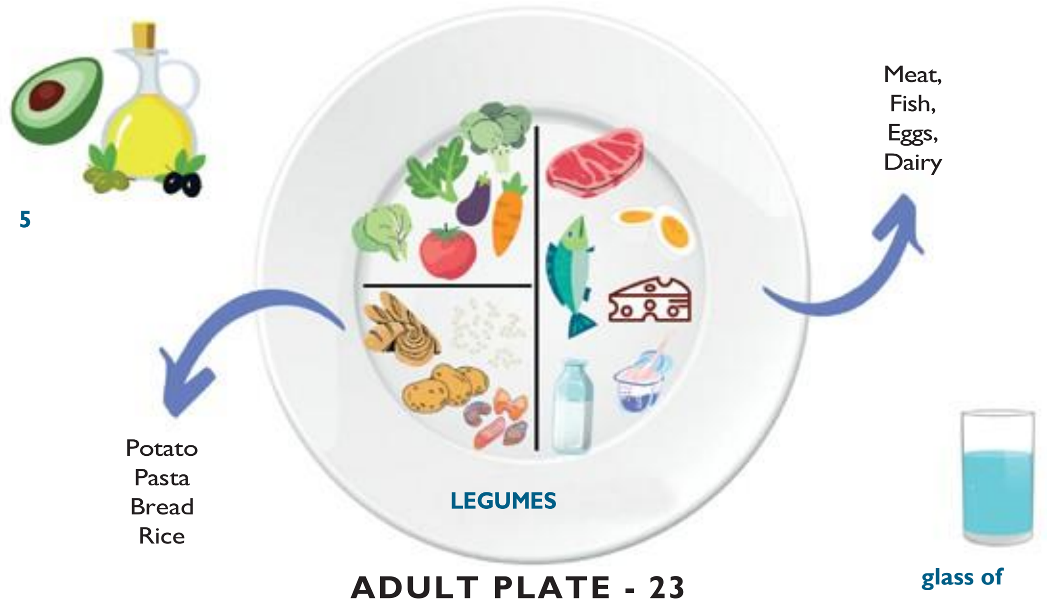

FIGURE 2 Plate method, adapted for oncology patients.
The plate sections show the recommended proportions of protein, carbohydrates, and vegetables. Legumes, having a composition, rich in protein, carbohydrates, and fiber, would have a versatile role in the diet of cancer patients.

- CHEESE: melted (in omelets, vegetable purees, etc.), grated (in soups, creams, purees, pasta, omelets, soufflés, crepes, etc.), chopped in salads or sandwiches.
- YOGURT: It is used in the preparation of fruit smoothies, in breakfasts with cereals, and is also often added to salad dressings.It can be enriched with powdered milk.
- EGG: chopped in salads, soups or vegetables, beaten in mashed potatoes, soups, creams, sauces, milkshakes or ice cream.You can add beaten egg whites or whole beaten egg to vegetable tart fillings, béchamel sauce, etc., and also egg whites to desserts such as flan or custard.
- MEAT AND FISH: chopped into vegetable dishes, salads, stews, sauces or soups. As a filling in tortillas, baked potatoes, eggplants, zucchini, etc., in stews, legume stews, etc.
- OILS AND FATS: the main fat used will be extra virgin olive oil (EVOO) in addition to cream, milk cream and butter, homemade mayonnaises added to purees of all kinds (vegetables, cereals, meat, fish, egg).Use 1-2 tablespoons of EVOO in each meal (breakfast, lunch and dinner)
- FRUITS AND DRIED FRUITS: walnuts, almonds, pistachios, pine nuts, chopped cashews, as a complement in sauces, stews and salads.

PHYSICAL EXERCISE AND MUSCLE RECOVERY PROGRAM.

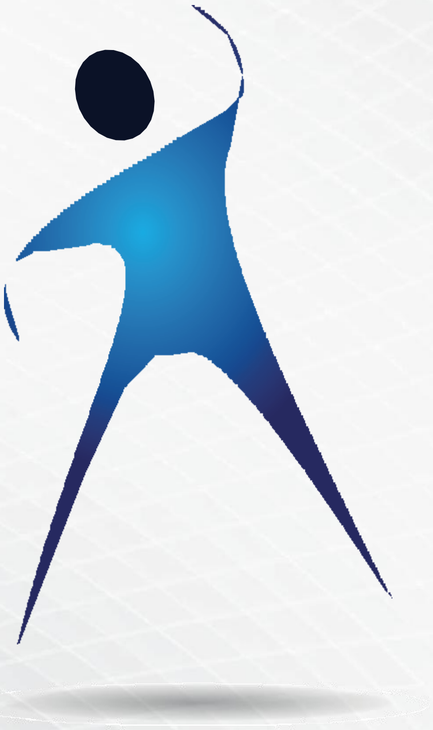

Prepared by:
Dr. Adela Gómez González
Rehabilitation and Physical Medicine Clinical Management Unit

Dr. José Manuel García Almeida
Endocrinology and Nutrition Unit

Virgen de la Victoria University Hospital – Malaga

AB-ProgamaDRE-100x180-230216-V6.indd 3/17/16 16:09

Physical Exercise Program

Warm-up phase:

Move your joints gently (neck, arms, waist and legs).Walk around the site for 5 minutes.

Aerobic training phase or endurance training:

Increased muscle endurance, cardiovascular benefits, and improved quality of life due to increased tolerance to physical exercise and ADLs.

- Pedal exerciser (use of arms and
- legs) Stationary bike
- Walks on flat terrain

15-30 minutes/day
(tolerable pace, without reaching fatigue)

Muscle strengthening phase:

Increased muscle mass, increased muscle strength, and improved performance of daily activities.

- 2-3 sessions / week.
- 3 sets of 5 repetitions of the exercise with 2-5 minutes of rest between sets.
- Intensity: progressive until fatigue is reached (bands, weights, bars...).
- Session duration:30-45 minutes.

AB-ProgamaDRE-100x180-230216-V6.indd 6-77/3/16 16:09

Strength exercises upper member

Involved in daily activities such as lifting objects, handling...

Shoulder press

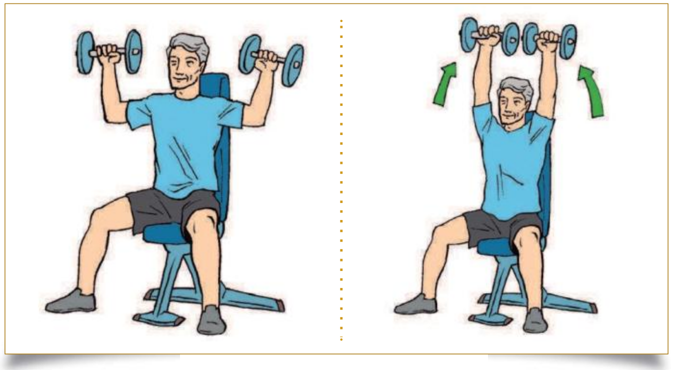

Rowing

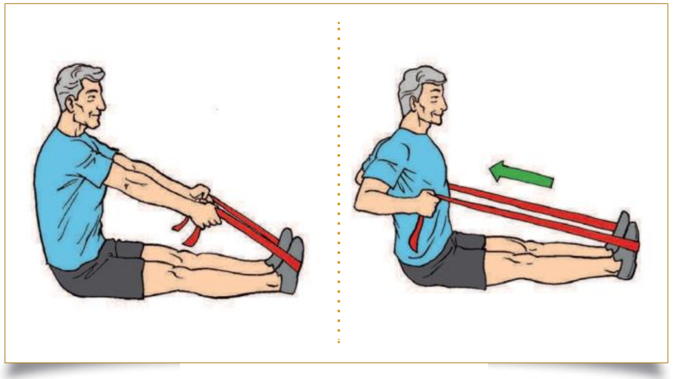

Arm extension

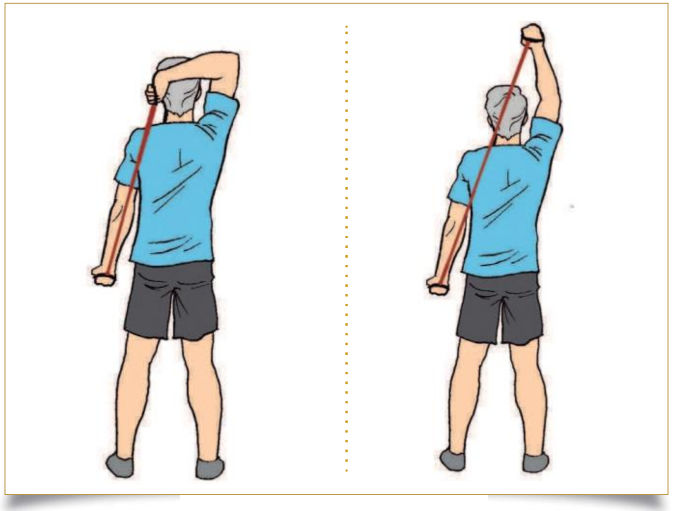

Push-up

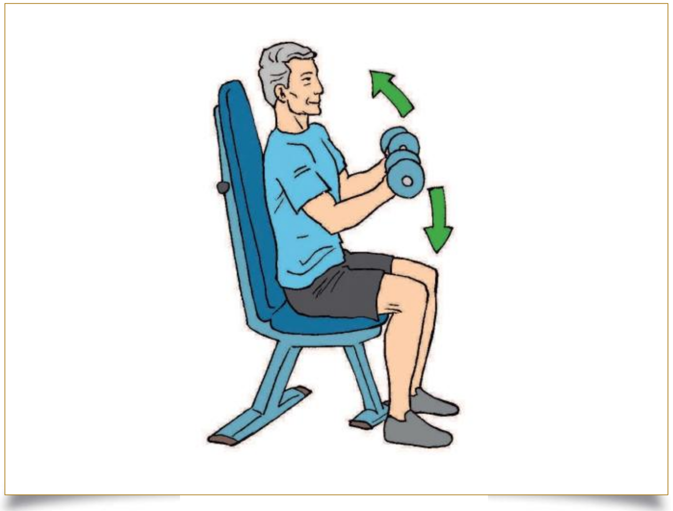

.

Strength exercises trunk and lower limb

They participate in standing and walking:

Trunk extension

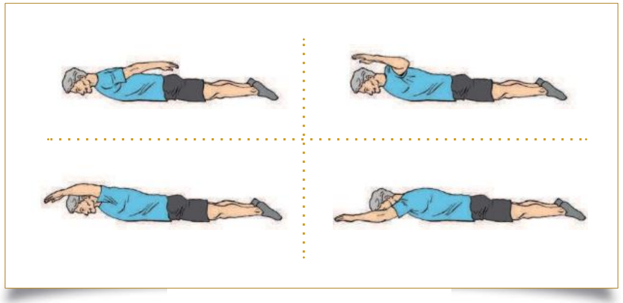

Abdominal exercises

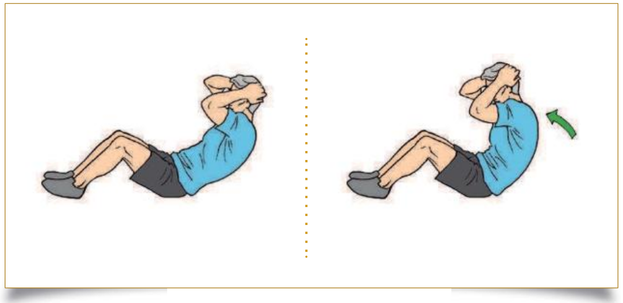

Squats

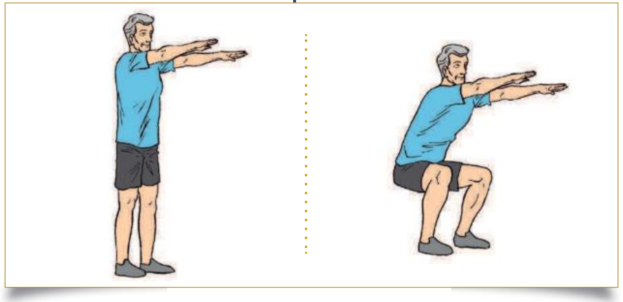

Leg extension

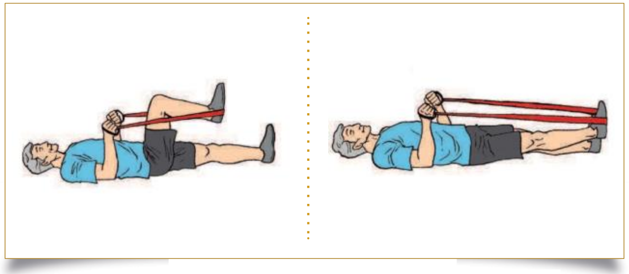

Knee extension

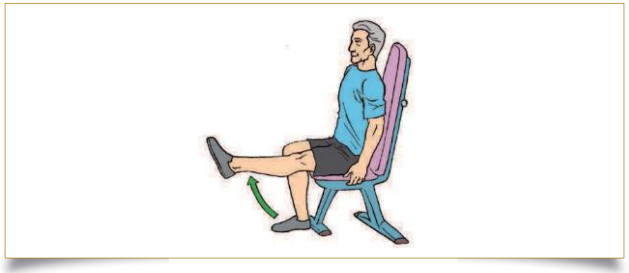

Knee injury

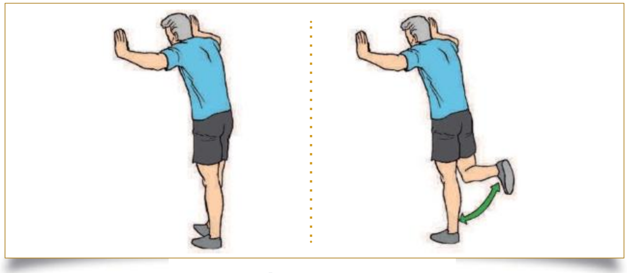

Lace

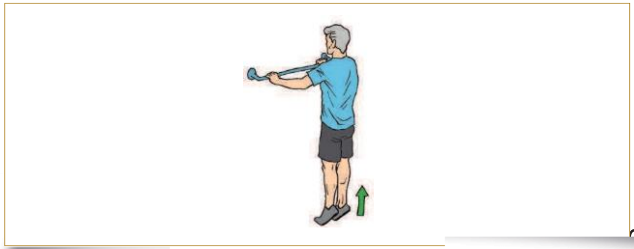

Supplement: Supplementary file 1 [file nutrients-18-01398-s001.zip › Supplementary material File S3. Dietary recommendations and physicial exercise v2.0.pdf]
